# Differential Substrate Usage and Metabolic Fluxes in *Francisella tularensis* Subspecies *holarctica* and *Francisella novicida*

**DOI:** 10.3389/fcimb.2017.00275

**Published:** 2017-06-21

**Authors:** Fan Chen, Kerstin Rydzewski, Erika Kutzner, Ina Häuslein, Eva Schunder, Xinzhe Wang, Kevin Meighen-Berger, Roland Grunow, Wolfgang Eisenreich, Klaus Heuner

**Affiliations:** ^1^Department of Chemistry, Chair of Biochemistry, Technische Universität MünchenGarching, Germany; ^2^Working Group “Cellular Interactions of Bacterial Pathogens”, ZBS 2, Robert Koch InstituteBerlin, Germany; ^3^Centre for Biological Threats and Special Pathogens, Division 2 (ZBS 2), Highly Pathogenic Microorganisms, Robert Koch InstituteBerlin, Germany

**Keywords:** *Francisella*, intracellular bacteria, ^13^C-labeling, isotopolog profiling, metabolic adaptation, tularemia, metabolic virulence

## Abstract

*Francisella tularensis* is an intracellular pathogen for many animals causing the infectious disease, tularemia. Whereas *F. tularensis* subsp. *holarctica* is highly pathogenic for humans, *F. novicida* is almost avirulent for humans, but virulent for mice. In order to compare metabolic fluxes between these strains, we performed ^13^C-labeling experiments with *F. tularensis* subsp. *holarctica* wild type (beaver isolate), *F. tularensis* subsp. *holarctica* strain LVS, or *F. novicida* strain U112 in complex media containing either [U-^13^C_6_]glucose, [1,2-^13^C_2_]glucose, [U-^13^C_3_]serine, or [U-^13^C_3_]glycerol. GC/MS-based isotopolog profiling of amino acids, polysaccharide-derived glucose, free fructose, amino sugars derived from the cell wall, fatty acids, 3-hydroxybutyrate, lactate, succinate and malate revealed uptake and metabolic usage of all tracers under the experimental conditions with glucose being the major carbon source for all strains under study. The labeling patterns of the *F. tularensis* subsp. *holarctica* wild type were highly similar to those of the LVS strain, but showed remarkable differences to the labeling profiles of the metabolites from the *F. novicida* strain. Glucose was directly used for polysaccharide and cell wall biosynthesis with higher rates in *F. tularensis* subsp. *holarctica* or metabolized, with higher rates in *F. novicida, via* glycolysis and the non-oxidative pentose phosphate pathway (PPP). Catabolic turnover of glucose *via* gluconeogenesis was also observed. In all strains, Ala was mainly synthesized from pyruvate, although no pathway from pyruvate to Ala is annotated in the genomes of *F. tularensis* and *F. novicida*. Glycerol efficiently served as a gluconeogenetic substrate in *F. novicida*, but only less in the *F. tularensis* subsp. *holarctica* strains. In any of the studied strains, serine did not serve as a major substrate and was not significantly used for gluconeogenesis under the experimental conditions. Rather, it was only utilized, at low rates, in downstream metabolic processes, e.g., *via* acetyl-CoA in the citrate cycle and for fatty acid biosynthesis, especially in the *F. tularensis* subsp. *holarctica* strains. In summary, the data reflect differential metabolite fluxes in *F. tularensis* subsp. *holarctica* and *F. novicida* suggesting that the different utilization of substrates could be related to host specificity and virulence of *Francisella*.

## Introduction

*Francisella tularensis* (*Ft*) is an intracellular Gram-negative pathogen that causes tularemia in many animals including humans (Ellis et al., 2002; Sjostedt, 2011). *Ft* has a broader host range order than any other known zoonotic pathogenic bacterium and causes the life-threatening disease in approximately 250 wildlife species including mammals, rodents, ticks and other anthropods (Foley and Nieto, 2010; Santic et al., 2010). Transmission mostly occurs *via* aerosol ingestion or skin inoculation. Due to the high pathogenicity of some strains against humans, *F. tularensis* is registered as a biological weapon (Dennis et al., 2001). Doses of as low as 10–20 bacteria of *Ft* subsp. *tularensis* (*Ftt*, Ft-type A, mainly found in North America) are infective and can result in lethal tularemia (Ellis et al., 2002). *Ft holarctica* (*Fth*, Ft-type B) is still highly infectious and found throughout the Northern hemisphere. Also in Europe, this strain is typically found in infected animals and humans. The bacterium *F. novicida* has been classified as a separate species, *F. novicida* (*Fno*) or as a *F. tularensis* subspecies *novicida* (Busse et al., 2010; Johansson et al., 2010; Kingry and Petersen, 2014). Here, we will utilize the nomenclature *Fno*.

The Gram-negative *Ftt* and *Fth* are specialized to replicate in the cytosol of host cells, especially of macrophages (for reviews, see Santic et al., 2006; Sjostedt, 2006; Keim et al., 2007; Jones et al., 2012). After engulfment of the pathogens into the macrophages, they form *Francisella*-containing phagosomes (FCPs) and trigger signals for the induction of the *Francisella* pathogenicity island (FPI). FPI also encodes a Type 6 secretion system (T6SS) (Chong et al., 2008; Clemens et al., 2015; Rigard et al., 2016) which finally leads to the escape of *Ft* into the cytosol of the host cell. For successful replication in this niche, the bacteria need to efficiently utilize nutrients from this environment, e.g., for energy generation and biosynthesis purposes. However, the cytosol of a host cell is not a nutrient-rich habitat in which every microorganism is able to grow, as show by injection experiments of e.g., *Salmonella typhimurium* (Goetz et al., 2001). Some intracellular bacteria including *Francisella, Listeria monocytogenes, Shigella*, and *Rickettsia* spp. have managed to exploit the limited nutrient supply of the cytosol for multiplication (Santic and Abu Kwaik, 2013). To efficiently utilize carbon substrates from the cytosolic compartment of the host cell, *L. monocytogenes* has been shown to take advantage from multiple substrates (e.g., glucose phosphate, glycerol and amino acids) which are shuffled in a bipartite metabolic network to serve specific metabolic traits (Grubmüller et al., 2014). Multiple substrate usage might be a general strategy for intracellular bacteria growing in the cytosol, but also in phagosomal compartments (Abu Kwaik and Bumann, 2013, 2015; Schunder et al., 2014; Eisenreich and Heuner, 2016; Gillmaier et al., 2016; Häuslein et al., 2016).

**Figure 1:**
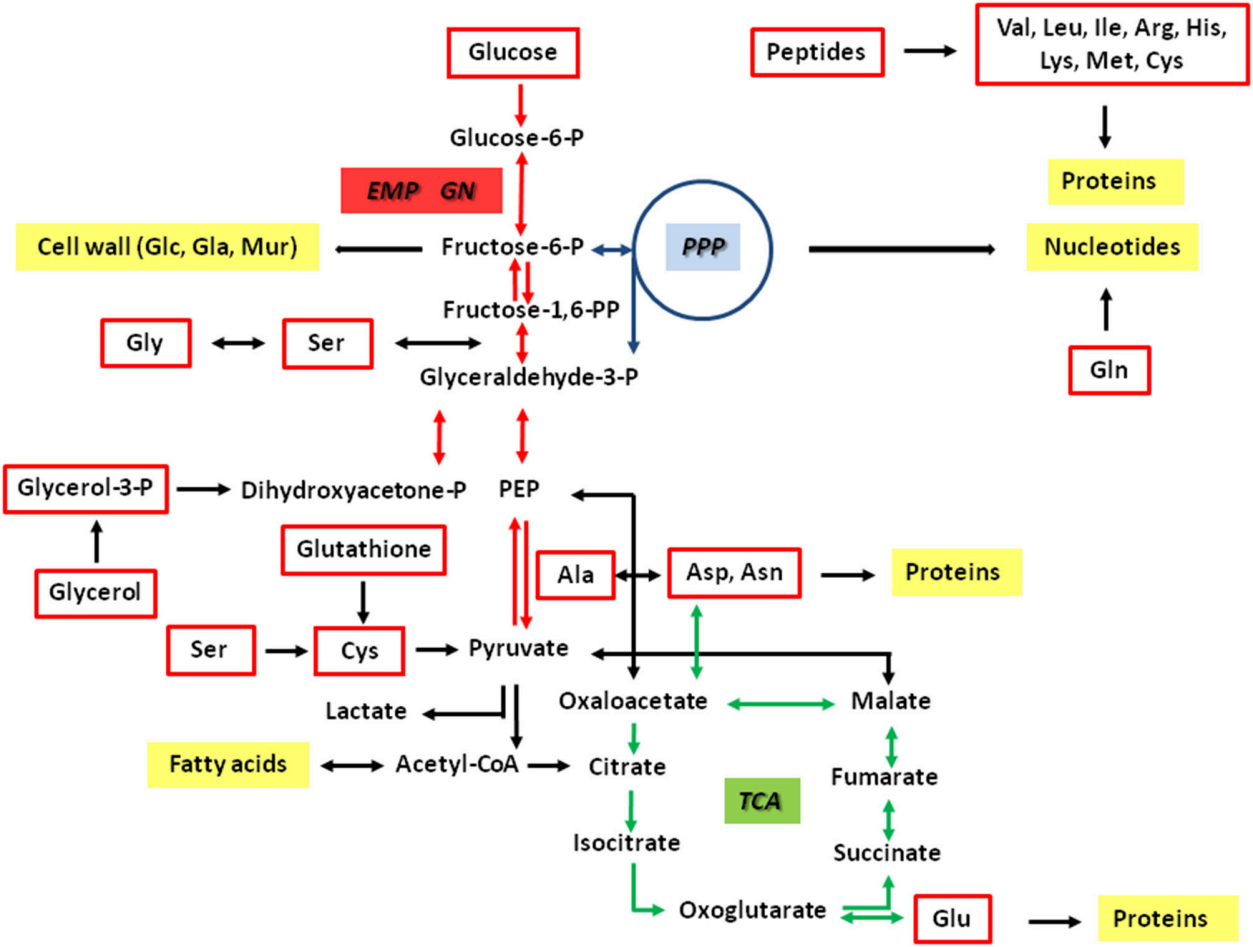
Hypothetical metabolic network of *Francisella tularensis*. The pathways are predicted on the basis of the genome sequences and earlier biochemical studies (Gyuranecz et al., 2010; Meibom and Charbit, 2010; Raghunathan et al., 2010; Barel et al., 2015). Potential carbon substrates are indicated by red boxes. Major metabolic products are indicated by yellow boxes. Reactions of the glycolytic pathway (EMP) and gluconeogensis (GN) are indicated by red arrows. Reactions of the pentose phosphate pathway (PPP) and the citrate cycle (TCA) are indicated by blue and green arrows, respectively. The interconnection between Gly and Ser includes the glycine cleavage system.

In case of *Francisella*, the relationship between nutrient usage and the *in vivo* life cycle is still poorly understood (Checroun et al., 2006; Meibom and Charbit, 2010; Santic and Abu Kwaik, 2013; Barel et al., 2015). It is current evidence that *Francisella* can also exploit some of the metabolic traits described earlier for *Listeria*. It was demonstrated that multiple substrates including amino acids (e.g., serine, glycine, cysteine, glutamate, glutamine, asparagine), small peptides and other gluconeogenetic substrates like glycerol or glycerol phosphate can also serve as carbon sources for intracellular *Ft* (Alkhuder et al., 2009; Raghunathan et al., 2010; Brown et al., 2013; Gesbert et al., 2013, 2015; Barel et al., 2015; Brissac et al., 2015; Ramond et al., 2015) (Figure 1). In agreement with this observation is the reported auxotrophy of *Ft* for several amino acids (e.g., for the branched chain amino acids, arginine, histidine, lysine, methionine and cysteine) and the apparent presence of several MFS-type and APC-type uptake systems for amino acids as seen in the genomes of *Ft* strains (Alkhuder et al., 2009; Meibom and Charbit, 2010). Some of these transporters were characterized in detail only recently (Gesbert et al., 2014, 2015; Ramond et al., 2014, 2015). In particular, serine was shown to be important for intracellularly multiplying *Francisella* strains (Meibom and Charbit, 2010; Raghunathan et al., 2010; Barel et al., 2012; Gesbert et al., 2013, 2015; Brown et al., 2014), which was also corroborated by a report showing that the SLC family of host amino acid transporters is important for intracellular replication of *Fth* strain LVS (Barel et al., 2012). Furthermore, it was demonstrated that the glycine cleavage system and the glycine dehydrogenase are also important for replication in serine limiting environments and that a *gcvT* mutant strain is auxotroph for serine. The glycine cleavage system of *Ftt* is also required in a murine model of infection (Brown et al., 2014). In addition, *Ft* requires Ser for growth under *in vitro* conditions, although *Ft* is not auxotroph for Ser (Meibom and Charbit, 2010; Brown et al., 2014).

In another agreement with *L. monocytogenes*, the sequenced genomes of some *Francisella* also display homologies to glycerol and glycerol phosphate transporters. On this basis, glycerol (and its phosphate) could serve a potential nutrient source at least for some *Francisella* species and *Ft* strains. Indeed, glycerol was shown to be metabolized by *Ftt, F. hispaniensis, F. philomiragia, Ft* subsp. *mediasiatica* (Huber et al., 2010) and *Fno* (Brissac et al., 2015). However, all strains of *Fth* are reported to metabolize glycerol phosphate but not glycerol (Gyuranecz et al., 2010).

In contrast to *L. monocytogenes, Ft* does neither encode potential uptake systems for glucose phosphate such as UhpT, nor PTS-type or non-PTS-type transporters of glucose. Despite this apparent lack of glucose or glucose phosphate uptake systems, glucose was shown to be metabolized by some *Francisella* strains. All of the *Fth* isolates were able to utilize glucose, whereas no strain could metabolize glucose 6-phosphate (Gyuranecz et al., 2010; Gesbert et al., 2014). In contrast to *Fth, Fno* could use cellobiose, galactose, and sucrose (Huber et al., 2010). On the basis of the genomes, all of these sugars can be degraded by the glycolytic pathway (EMP pathway) and/or the non-oxidative pentose phosphate pathway (PPP), but not *via* the Entner-Doudoroff pathway (ED) and the oxidative branch of the PPP. Further downstream, a complete citrate cycle (TCA) appears to be important to provide NADH for energy production. Anaplerotic reactions could interconnect the EMP with the TCA. A schematic overview of the key metabolic pathways is given in Figure 1.

These metabolic capacities already point at a highly versatile and maybe species- and strain-dependent substrate usage of *Ft*. Interestingly, the genetic analysis of the *Ftt* Schu S4 genome revealed an unusually high amount of disrupted biosynthetic pathways which could indicate that *Ftt* is an obligate host-dependent bacterium in its natural life cycle (Larsson et al., 2005; Meibom and Charbit, 2010). However, the details of metabolic fluxes in *Ft* are still rather obscure. It is not known whether the usage of substrates is related to virulence of different *Francisella* species. In order to shed light onto this item and to study the metabolism of *Ft* in general, we applied comparative isotopolog profiling experiments with a highly pathogenic *Fth* strain recovered recently from a beaver (Schulze et al., 2016), an attenuated live vaccine strain (LVS) of *Fth*, and the (for humans) almost apathogenic *Fno* strain U112 (Larson et al., 1955; Hollis et al., 1989; Clarridge et al., 1996). Thereby, a rather detailed view into the metabolic network of *Fth* and *Fno* could be made with some remarkable metabolic differences between *Fth* and *Fno*, suggesting that substrate usage and metabolic fluxes could also trigger pathogenesis of different *Francisella* species.

## Materials and methods

### Strains, growth conditions, media and buffers

Strains used in this study were *Fno* strain U112 (ATCC 15482; Larson et al., 1955), *Fth* strain LVS (ATCC 29684) and *Fth* wild type strain (“Beaver” isolate; Ft-type B) (Schulze et al., 2016). *Francisella* strains were cultivated in medium T (Pavlovich and Mishan'kin, 1987; Becker et al., 2016) containing 1% brain heart infusion broth (Difco Laboratories, Inc., Sparks, MD, USA), 1% bacto tryptone (Difco), 1% technical casamino acids (Difco), 0.005 g of MgSO_4_, 0.01% FeSO_4_, 0.12% sodium citrate, 0.02% KCl, 0.04% K_2_HPO_4_, 0.06% L-cysteine and 1.5% glucose at 37°C.

### Labeling experiments of *Francisella* strains

1 L of growth medium (medium T) was supplemented with 2 g of [U-^13^C_6_]glucose (11 mM), 2 g of [1,2-^13^C_2_]glucose (11 mM), 0.3 g of [U-^13^C_3_]Ser (3 mM), or 2.5 g of [U-^13^C_3_]glycerol (25 mM), respectively. Volumes of 250 ml of supplemented medium T were inoculated with 2–4 ml of an over-night culture of the *Francisella* strains. Incubation was carried out at 37°C and 220 rpm. The optical density at 600 nm (OD_600_) was determined at regular intervals. An OD_600_ of ~1.8 correlated with stationary growth. Cultures in medium T reached stationary growth at 26 h. Before harvesting, a culture aliquot was plated onto lysogeny broth (LB) agar (Bertani, 1951, 2004) to rule out the possibility of contamination. The bacteria were pelleted at 4,700 g and 4°C for 15 min. The supernatant was discarded and the bacterial pellet was autoclaved at 120°C for 20 min. Then, the pellet was resuspended in 3 ml of water and lyophilized.

### Analysis of medium T

#### Determination of free amino acids

1 mL of autoclaved medium T was dried under N_2_ flux. The residue was suspended in 1 mL of methanol and was centrifuged (10,000 g for 20 min, 4°C). 5 μL of a 10 mM norvaline solution (internal standard) was added to the supernatant. The mixture was then dried under N_2_ flux. The residue was treated with 50 μL of N-(tert-butyldimethylsilyl)-N-methyl-trifluoroacetamide containing 1% tert-butyldimethylsilylchloride and 50 μL of water free acetonitrile at 70°C for 1 h. The TBDMS-derivatives of amino acids were then quantified by GC-MS.

#### Determination of protein-bound amino acids

50 μL of autoclaved medium T was dried under N_2_ flux. The residue was suspended in 0.5 mL of 6 M hydrochloric acid and hydrolyzed for 15 h at 105°C. The hydrolysate was dried under N_2_ flux. The residue was suspended in 200 μL of 50% acetic acid using an ultrasonic bath for 3 min. The solution was centrifuged and the supernatant was applied onto a small column of Dowex 50W X8 (7 × 10 mm; 200–400 mesh, 34–74 μm, H^+^-form). The column was first washed with 2 mL H_2_O, then eluted with 1 mL 4 M aqueous ammonia. 5 μL of 10 mM norvaline solution (internal standard) was added to the eluate and the mixture was dried under N_2_ flux at 55°C. The residue was treated with 50 μL of N-(tert-butyldimethylsilyl)-N-methyl-trifluoroacetamide containing 1% tert-butyldimethylsilylchloride and 50 μL of water free acetonitrile at 70°C for 30 min. The TBDMS-derivatives of amino acids were then quantified by GC-MS.

#### Determination of free glucose and glucose from acid-labile polysaccharides

100 μL of 10 mM fructose solution (internal standard) was added to 1 mL of autoclaved medium T, the mixture was dried under N_2_ flux. 1 mL of a solution of acetone containing 2% sulfuric acid was added. After 1 h at room temperature, 2 mL of saturated NaCl solution and 2 mL of saturated NaCO_3_ solution were added. The reaction mixture was extracted two times with 3 mL of ethyl acetate. The organic phase was collected and dried under N_2_ flux. The residue was reacted with 100 μL of acetyl anhydride at 60°C in 100 μL of water free ethyl acetate for 16 h. The reaction mixture was dried under N_2_ flux, dissolved in 100 μL of water free ethyl acetate and subjected to GC-MS analysis.

### Workup of *Francisella* cells

#### Aqueous cell extraction and analysis of free fructose

About 30 mg of bacterial sample (lyophilized cell pellet) and 500 μL of glas beads (0.25–0.5 mm) were suspended in 1 mL of H_2_O. The mixture was subjected to mechanical disruption using a ribolyser system (6.5 s^−1^, 20 s, 27°C, three times). The mixture was centrifuged (10,000 g for 20 min, 4°C) and the supernatant was then dried under N_2_ flux. The residue was treated with 100 μL of methoxamine hydrochloride pyridine solution (40 mg/mL) at 30°C for 90 min. The reaction mixture was dried under N_2_ flux. Finally, the residue was treated with 100 μL of N-methyl-N-trifluoroacetamide at 37°C for 45 min and subjected to GC-MS analysis.

#### Methanolic cell extraction and analysis of polar metabolites

About 30 mg of bacterial sample (lyophilized cell pellet) and 500 μL of glass beads (0.25–0.5 mm) were suspended in 1 mL of methanol. The mixture was subjected to mechanical disruption using a ribolyser system (6.5 s^−1^, 20 s, 27°C, three times). The mixture was centrifuged (10,000 g for 20 min, 4°C) and the supernatant was then dried under N_2_ flux. The residue was treated with 50 μL of N-(tert-butyldimethylsilyl)-N-methyl-trifluoroacetamide containing 1% tert-butyldimethylsilylchloride and 50 μL acetonitrile at 70°C for 1 h. The resulting TBDMS-derivatives were then analyzed by GC-MS.

#### Total hydrolysis and analysis of protein-derived amino acids

About 2 mg of bacterial sample (lyophilized cell pellet) were suspended in 1 mL of 6 M hydrochloric acid and hydrolyzed for 15 h at 105°C. The reaction mixture was dried under N_2_ flux. The residue was suspended in 200 μL of 50% acetic acid using an ultrasonic bath for 3 min. The solution was centrifuged and the supernatant was applied onto a small column of Dowex 50W X8 (7 × 10 mm; 200–400 mesh, 34–74 μm, H^+^-form). The column was first washed with 2 mL H_2_O, then eluted with 1 mL 4 M aqueous ammonia. 5 μL of 10 mM norvaline solution (internal standard) was added to the eluate and the mixture was dried under N_2_ flux at 55°C. The residue was treated with 50 μL of N-(tert-butyldimethylsilyl)-N-methyl-trifluoroacetamide containing 1% tert-butyldimethylsilylchloride and 50 μL of water free acetonitrile at 70°C for 30 min. The TBDMS-derivatives of amino acids were then quantified by GC-MS (Eylert et al., 2010).

#### Hydrolysis of polysaccharides and analysis of glucose

About 5 mg of bacterial sample (lyophilized cell pellet) was hydrolyzed for 15 h with 0.5 mL of 3 M methanolic HCl at 80°C. The reaction mixture was centrifuged (10,000 g for 20 min, 4°C) and the supernatant was dried using a stream of dry N_2_ gas. 1 mL of a solution of acetone containing 2% sulfuric acid was added. After 1 h at room temperature, 2 mL of saturated NaCl solution and 2 mL of saturated NaCO_3_ solution were added. The reaction mixture was extracted two times with 3 mL of ethyl acetate. The organic phase was collected and dried under N_2_ flux. The residue was reacted with 100 μL of acetyl anhydride at 60°C in 100 μL of water-free ethyl acetate for 16 h. The reaction mixture was dried under N_2_ flux and resolved in 100 μL of water free ethyl acetate for GC-MS analysis.

#### Hydrolysis of cell wall and analysis of cell-wall sugars

About 15 mg of bacterial sample (lyophilized cell pellet) was hydrolyzed for 15 h in 0.5 mL of 6 M hydrochloric acid at 105°C. The reaction mixture was filtered and the filtrate was dried under N_2_ flux. The residue was treated with 100 μL hexamethyldisilazane at 120°C for 3 h. The resulting TMS-derivatives were analyzed by GC-MS.

### GC-MS and isotopolog analysis

All derivatives mentioned above were analyzed by GC-MS using a GCMS-QP 2010 Plus spectrometer (Shimadzu, Duisburg, Germany) as described earlier (Häuslein et al., 2016). All data were collected using LabSolution software *(Shimadzu)*. The samples were analyzed three times as technical replicates. The overall ^13^C excess (mol-%) and the relative contributions of isotopomers (%) were computed by an Excel-based in-house software package (Eylert et al., 2008) according to Lee et al. (1991).

#### Analysis of MEOX-TMS-fructose and TBDMS-derivatives of polar metabolite mixtures

The column was first developed at 100°C for 2 min, then using a gradient of 3°C min^−1^ to 234°C, followed by 1°C min^−1^ to 237°C and 3°C min^−1^ to 260°C. Finally, the column was heated at a gradient of 10°C min^−1^ to a final temperature of 320°C where it was hold for 2 min.

#### Analysis of TBDMS-amino acids

The column was first developed at 150°C for 3 min, then using a gradient of 7°C min^−1^ to 280°C where it was hold for 3 min.

#### Analysis of diisopropylidene/acetate derivatives of glucose

The column was first developed at 150°C for 3 min, then using a gradient of 10°C min^−1^ to 220°C, then by 50°C min^−1^ to 280°C where it was hold for 3 min.

#### Analysis of TMS-sugars

The column was first developed at 70°C for 5 min, then using a gradient of 5°C min^−1^ to 310°C where it was hold for 1 min.

## Results

### Experimental approach

To investigate the usages of possible substrates, i.e., of glucose, serine or glycerol, we performed labeling experiments with different *Francisella* strains growing in medium T in the presence of [U-^13^C_6_]glucose, [1,2-^13^C_2_]glucose, [U-^13^C_3_]serine, or [U-^13^C_3_]glycerol as tracers. More specifically, we supplemented the highly pathogenic *Fth* WT strain (beaver isolate) (Schulze et al., 2016), the *Fth* life vaccine strain LVS, or the less pathogenic *Fno* strain U112 with 11 mM [U-^13^C_6_]glucose, 11 mM [1,2-^13^C_2_]glucose, 3 mM [U-^13^C_3_]serine, or 25 mM [U-^13^C_3_]glycerol, respectively. In each of the labeling experiments, the strains were grown at 37°C for 26 h. The growth curves of *Fth* and *Fno* were apparently identical in each of the labeling experiments (data not shown). The cells were harvested at final optical densities (OD_600_) of approximately 1.8 (stationary phase). The bacteria were pelleted, autoclaved and lyophilized affording about 100 mg of dry cell pellet from a given experiment. From the dried cell pellets, polar metabolites including free fructose were extracted. Proteins, cell wall, or acid-labile polysaccharides were then hydrolyzed and amino acids, glucosamine, muramic acid and glucose were isolated from the respective hydrolysates. The isolated metabolites were converted into appropriate derivatives which were finally analyzed by GC/MS spectrometry (for details, see Materials and Methods). Each of the labeling experiments was done at least two times, in most of the settings three times (biological replicates). Each of the GC-MS samples was analyzed three times (technical replicates). On this basis, the data given below represent mean values and standard deviations using six to nine experimental values for each metabolite under study (see also Supplemental Tables S1–S27 for numerical data). This provided a robust basis for the comparative approach.

### Labeling experiments with [U-^13^C_6_]glucose

#### Isotopolog profiles of free fructose, glucose from acid-labile polysacharides, and amino sugars from the cell wall

**Figure 2:**
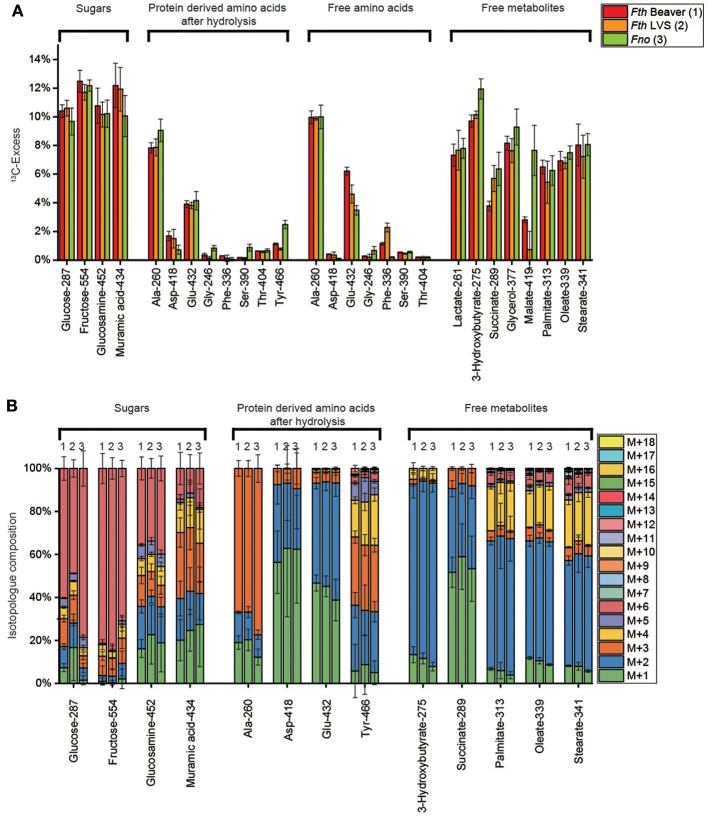
**(A)** ^13^C-Excess (mol%) and **(B)** the fractional isotopolog distributions (%) in key metabolites of *Francisella tularensis* subspecies *holartica* WT strain (beaver isolate) (*Fth* Beaver, 1), the *Francisella tularensis* subspecies *holartica* life vaccine strain LVS (*Fth* LVS, 2), or the less pathogenic *Francisella novicida* strain U112 (*Fno*, 3) grown in medium T supplied with 11 mM [U-^13^C_6_]glucose. ^13^C-Excess (mol%) and relative fractions of isotopologs (%) were determined by GC/MS of silylated derivatives at the indexed m/z values. Error bars indicate standard deviations from the means of 6 to 9 values 2–3 × biological replicates, 3 × technical replicates. M+1, M+2, M+3, etc. indicate isotopologs carrying 1, 2, 3, etc. ^13^C-atoms. For numerical values, see Supplemental Tables S1, S5, S9, S13, S17, S21.

Hexoses could be isolated in form of free fructose (isolated from the aqueous cell extracts), polysaccharide-derived glucose obtained by mild acidic hydrolysis and cell wall-derived amino sugars (i.e., glucosamine and muramic acid) by harsh acidic treatment. Each of the sugars displayed high ^13^C-enrichments (about 10–12%, Figure 2A) reflecting efficient uptake and usage of exogenous glucose by any of the *Fth* and *Fno* strains under study. The incorporation rates were slightly higher in the *Fth* strains (11.2 ± 0.9% averaged over the four hexoses) in comparison to *Fno* (10.5 ± 1.1%) (See also Supplemental Table S9). The major isotopolog in free fructose was M+6 (Figure 2B) by conversion of [U-^13^C_6_]glucose into [U-^13^C_6_]fructose probably *via* glucose 6-phosphate and fructose 6-phosphate. However, significant metabolic turnover in hexose metabolism (i.e., degradation to triose phosphates and subsequent reformation of hexoses) became evident on the basis of the detected M+1 to M+5 isotopologs in free fructose (20–30%) and glucose from the polysaccharides (20–50%) and even more pronounced in glucosamine and muramic acid from the cell wall (60–90%) (Figure 2B). The relative fractions of the isotopologs reflecting this glycolytic cycling were apparently similar for *Fth* and *Fno*, with the notable exception of polysaccharide-derived glucose from *Fno* showing significantly lower rates.

#### Isotopolog profiles of protein-derived amino acids

Harsh acidic hydrolysis of the bacterial pellet afforded a mixture of amino acids mostly (>90 %) derived from the protein fraction. By silylation, these amino acids were converted into TBDMS-derivatives that could be analyzed by GC/MS following our standard protocols. Since Trp and Cys were destroyed during the acidic hydrolysis, we could not obtain data for these amino acids. Asn and Gln were converted into Asp and Glu, respectively, and the reported values for Asp and Glu therefore represent averages for Asn/Asp and Gln/Glu, respectively. GC/MS analysis of 15 TBDMS-amino acids revealed ^13^C enrichments (and therefore *de novo* biosynthesis) in 7 amino acids from the *Fth* strains: Ala > Glu > Asp > Tyr > Thr > Phe > Gly = Ser (8–0.1%) and in *Fno*: Ala > Glu > Tyr >> Thr = Asp = Gly = Ser > Phe) (9–0.1%) (Figure 2A). His, Ile, Leu, Val, Lys, and Pro were apparently unlabeled (Supplemental Table S1) demonstrating that these amino acids were derived from unlabeled substrates (e.g., amino acids or peptides) present in brain heart infusion broth, bacto trypton and casamino acids of medium T (see also Supplemental Tables S25, S26).

In any of the detected amino acids, there were no significant differences in the overall ^13^C-enrichments (Figure 2A) and the isotopolog profiles as well (Figure 2B) between *Fth* LVS and the highly pathogenic beaver isolate of *Fth*. In contrast, significant differences could be noticed when comparing the *Fth* strains with *Fno*. More specifically, the ^13^C excess values from protein-derived amino acids (especially of Ala, Ser, and Tyr) using glucose as the substrate were higher in *Fno* than in the *Fth* strains. From the MS traces, the relative fractions of ^13^C-isotopologs in the ^13^C-enriched amino acids could be determined (Figure 2B). Again, the relative fractions of the isotopologs were highly similar when comparing the *Fth* strains, but significantly different for some amino acids when comparing *Fth* with *Fno*.

The high fractions of [U-^13^C_3_]-Ala (M+3) in the fragment containing all three carbon atoms of the original alanine molecule (Ala-260) indicated efficient usage of glucose present in the complex medium and the formation of [U-^13^C_3_]pyruvate by degradation of [U-^13^C_6_]glucose (see below). Genes encoding enzymes for the usage of glucose by glycolysis (EMP pathway) and the non-oxidative PPP are present in the genomes of the sequenced *Francisella* strains (Figure 1). Surprisingly, however, genes encoding alanine dehydrogenase and Glu/Asp transaminase converting [U-^13^C_3_]pyruvate into [U-^13^C_3_]-Ala are not annotated in the *Francisella* genomes. The lower relative amounts of ^13^C_2_- and ^13^C_1_-Ala can be explained by the expected pyruvate formation *via* Asp by an aspartate 4-decarboxylase as also reflected in the genomes (Figure 1). The observed ratio of ^13^C_2_- and ^13^C_1_-Ala in the global ^13^C-excess of Ala-260 suggested that approximately 35% of Ala were synthesized from Asp, and 65% from pyruvate in *Fth*, whereas the pyruvate contribution was slightly higher in *Fno* (about 75%). Subsequently, ^13^C from pyruvate could enter the TCA cycle mainly *via* [U-^13^C_2_]acetyl-CoA as reflected by the detected ^13^C_2_-Glu (M+2, Figure 2B) formed from ^13^C_2_-α-ketoglutarate, and ^13^C_2_-Asp (M+2) formed from ^13^C_2_-oxaloacetate. The labeling profiles of Tyr were more complex and contained a large fraction of M+4 that could be explained by the shikimate/chorismate pathway using [U-^13^C_4_]erythrose 4-phosphate as a precursor which could be synthesized *via* the PPP (forming [U-^13^C_4_]erythrose 4-phosphate from [U-^13^C_6_]fructose 6-phosphate by the transketolase reaction). The M+2 and M+3 fraction could arise by incorporation of [U-^13^C_3_]PEP, one of which becomes decarboxylated during the chorismate route.

#### Isotopolog profiles of free polar metabolites

In the aqueous and methanolic cell extracts, we focused on about 20 metabolites including free amino acids, lactate, glycerol, succinate, malate, and fatty acids. Generally, the ^13^C-enrichments and isotopolog profiles of free amino acids resembled those from the protein-bound amino acids (Figure 2A). The ^13^C-excess values and isotopolog compositions of lactate and glycerol resembled that of alanine indicating a *quasi*-equilibrium state of isotope distribution between these related metabolites.

Fatty acids and 3-hydroxybutyrate were also highly ^13^C-enriched (6–12%) suggesting efficient usage of [U-^13^C_6_]glucose for fatty acids biosynthesis by glucose degradation leading to [U-^13^C_2_]acetyl-CoA precursors. Carbon flux *via* [U-^13^C_2_]acetyl-CoA into the TCA cycle is also seen in the labeling profiles of succinate and malate with high fractions of M+2 (like in the TCA-derived amino acid aspartate). As shown in Figure 2, the values for the metabolites from *Fth* or *Fno* were again very similar, with a slight tendency of higher incorporation into metabolites from *Fno*.

In total, the labeling patterns of multiple metabolites obtained from the labeling experiment with 11 mM [U-^13^C_6_]glucose suggested its efficient uptake and direct usage for polysaccharide synthesis, but also its degradation *via* the glycolytic pathway to pyruvate/Ala and, at lower rates, to acetyl-CoA as a precursor for fatty acids or as a substrate for the TCA cycle. Moreover, the observed complex isotopolog compositions in cell wall sugars, glucosamine and muramic acid, indicated direct incorporation of the intact hexose tracer, but also glycolytic turnover e.g., *via* the PPP and/or gluconeogenesis. In comparison of the strains, usage of glucose appears to be more directed for cell wall synthesis in the *Fth* strains, whereas degradation and downstream usage seems to be more pronounced in *Fno*.

### Labeling experiments with [1,2-^13^C_2_]glucose

**Figure 3:**
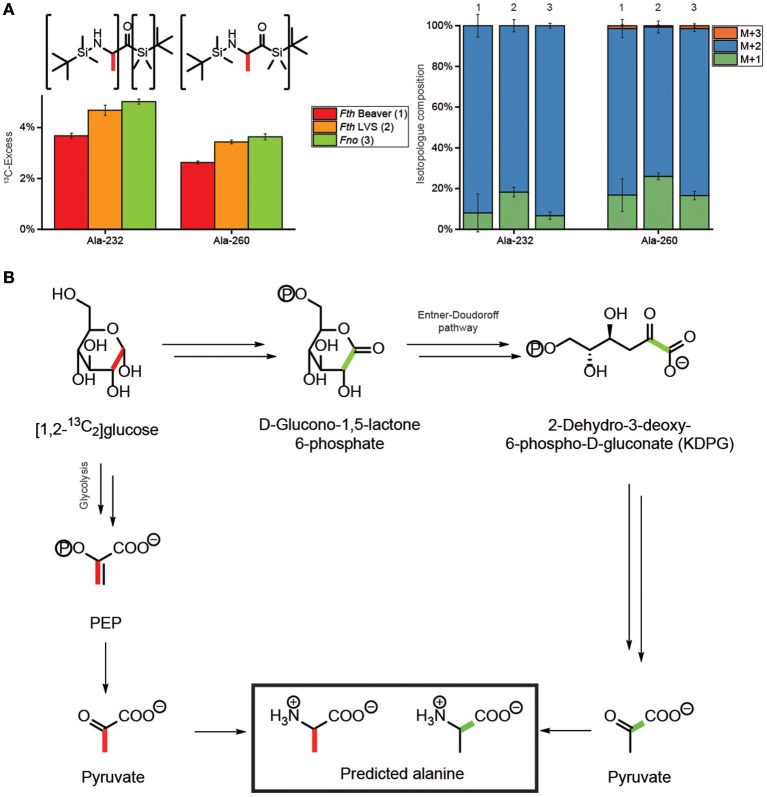
Transfer of ^13^C-label from [1,2-^13^C_2_]glucose into alanine of *Fth* Beaver (1), *Fth* LVS (2), or *Fno* (3). **(A)** ^13^C-Excess (mol%) and the fractional isotopolog distributions (%) in the silylated fragments Ala-260 and Ala-232 carrying C1-C3 and C2-C3 of the original alanine carbon chain, respectively. **(B)** Conversion of [1,2-^13^C_2_]glucose into pyruvate and alanine *via* glycolysis (EMP) (left, with red bars indicating the ^13^C-labels) or the Entner-Doudoroff pathway (ED) (right, with green bars indicating the ^13^C-labels). The observed label distribution with ^13^C at C2 and C3 (indicated by red bars in panel **A**), only matches the predicted pattern *via* the EMP in panel **(B)**. For more details, see also text.

Since the labeling profiles in metabolites from [U-^13^C_6_]glucose could not discriminate between glucose degradation *via* glycolysis, the PPP or the Entner-Doudoroff pathway, we performed additional experiments with [1,2-^13^C_2_]glucose. As a key analyte to distinguish between these routes, we had a detailed look at Ala which displayed in the mass spectrum of its TBDMS-derivative a fragment at m/z of 232 which is devoid of C-1 (Figure 3A). In comparison to the mass trace at m/z of 260 showing all three carbon atoms of the original alanine molecule, some positional ^13^C-assignment can be made on this basis. As expected, the Ala-derivative carrying all three C-atoms (Ala-260) displayed high fractions of M+2. The fragment with the smaller mass (Ala-232) carrying C-2 and C-3 of Ala showed a similarly high fraction for M+2 which is only possible when C-1 of Ala was not ^13^C-labeled (Figure 3A). It can therefore be concluded that ^13^C from [1,2-^13^C_2_]glucose was predominantly, if not entirely, transferred into the positions 2 and 3 of Ala (Figure 3A). This pattern excludes the Entner-Doudoroff pathway, since cleavage of [1,2-^13^C_2_]glucose would then lead to [1,2-^13^C_2_]pyruvate and [1,2-^13^C_2_]Ala finally affording M+1 in the Ala-232 fragment, but not M+2 (Figure 3B). However, glycolysis yields [2,3-^13^C_2_]pyruvate/Ala from [1,2-^13^C_2_]glucose, in line with our experimental data (Figure 3B). Degradation of [1,2-^13^C_2_]glucose *via* the PPP would results in single labeled or unlabeled triose phosphates and subsequently pyruvate/Ala. On this basis, a major contribution of the PPP in the formation of the precursors of Ala can also be excluded. Thus, glycolysis (EMP pathway) is the predominant route for glucose usage in *Francisella*.

### Labeling experiments with [U-^13^C_3_]serine

**Figure 4:**
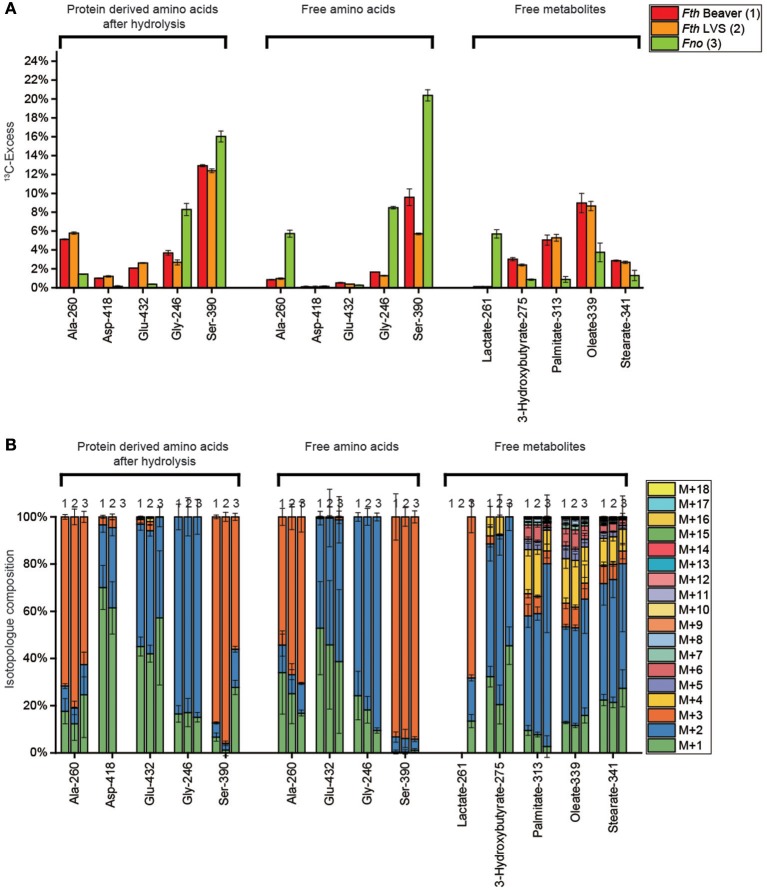
**(A)** ^13^C-Excess (mol%) and **(B)** the fractional isotopolog distributions (%) in key metabolites of *Fth* Beaver (1), *Fth* LVS (2), or *Fno* (3) grown in medium T supplied with 3 mM [U-^13^C_3_]serine. For numerical values, see Supplemental Tables S3, S7, S11, S15, S19, S23. For more details, see also legend to Figure 2.

Using our experimental system, we showed that exogenous 3 mM [U-^13^C_3_]serine is taken up by *Fth* and *Fno* as reflected by the ^13^C-enrichments in serine isolated from the methanolic cell extracts (*Fth* beaver isolate, 10%; *Fth* LVS, 6%, *Fno*, 20%) (See also Supplemental Table S7) (Figure 4A). The usage of serine for protein biosynthesis was also gleaned from the ^13^C-enrichments in protein-bound Ser (*Fth* strains, 12%; *Fno*, 16%) (See also Supplemental Table S3). The predominant isotopologs in any of the serine samples were the M+3 species indicating less metabolic turnover leading to M+1 or M+2 serine (Figure 4B).

However, the metabolic degradation of serine became evident from the detected ^13^C-enrichments in a set of metabolites including glycine (Figure 4A) that can be derived from serine by a hydroxymethytransferase. Amino acids derived from pyruvate (Ala) and the TCA (Asp and Glu), fatty acids (derived from acetyl-CoA), and some polar metabolites directly related to pyruvate (lactate from the *Fno* strain) or fatty acid metabolism (3-hydroxybutyrate) also acquired significant ^13^C-label from serine (Figure 4A). However, amino acids derived from the PPP (Phe and Tyr) and any of the analyzed sugars (free fructose, glucose from glycogen and amino sugars from the cell wall) were apparently unlabeled (Supplemental Tables S3, S7, S11) suggesting that serine is not serving as a glucogenic substrate, under our experimental conditions.

Compared to the *Fth* strains, *Fno* exhibited a different pattern of ^13^C-excess with [U-^13^C_3_]-Ser as a substrate. In the *Fth* strains, there was more flux from Ser directly into the biosynthesis of proteins (with the exception of glycine), *via* the precursors, pyruvate and oxaloacetate used for amino acid biosynthesis, and into acetyl-CoA used for fatty acid biosynthesis. In *Fno*, however, the free amino acids Ala, Ser and Gly, as well as lactate acquired much more (> factor 3) ^13^C-label from [U-^13^C_3_]-Ser. Interestingly, in *Fth* there was less ^13^C flux from Ser to Gly, than from Ser to Ala, in *Fno* it was *vice versa* (Figure 4A). Probably, in *Fth*, Ser is more effectively used as a substrate during the exponential phase of growth with high rates of protein and lipid biosynthesis, but less in post-exponential or stationary phase of growth (sampling point), as reflected by the lower enrichments in the free short living metabolites, probably because no further ^13^C-Ser was available at this late growth phase of *Fth*.

### Labeling experiments with [U-^13^C_3_]glycerol

**Figure 5:**
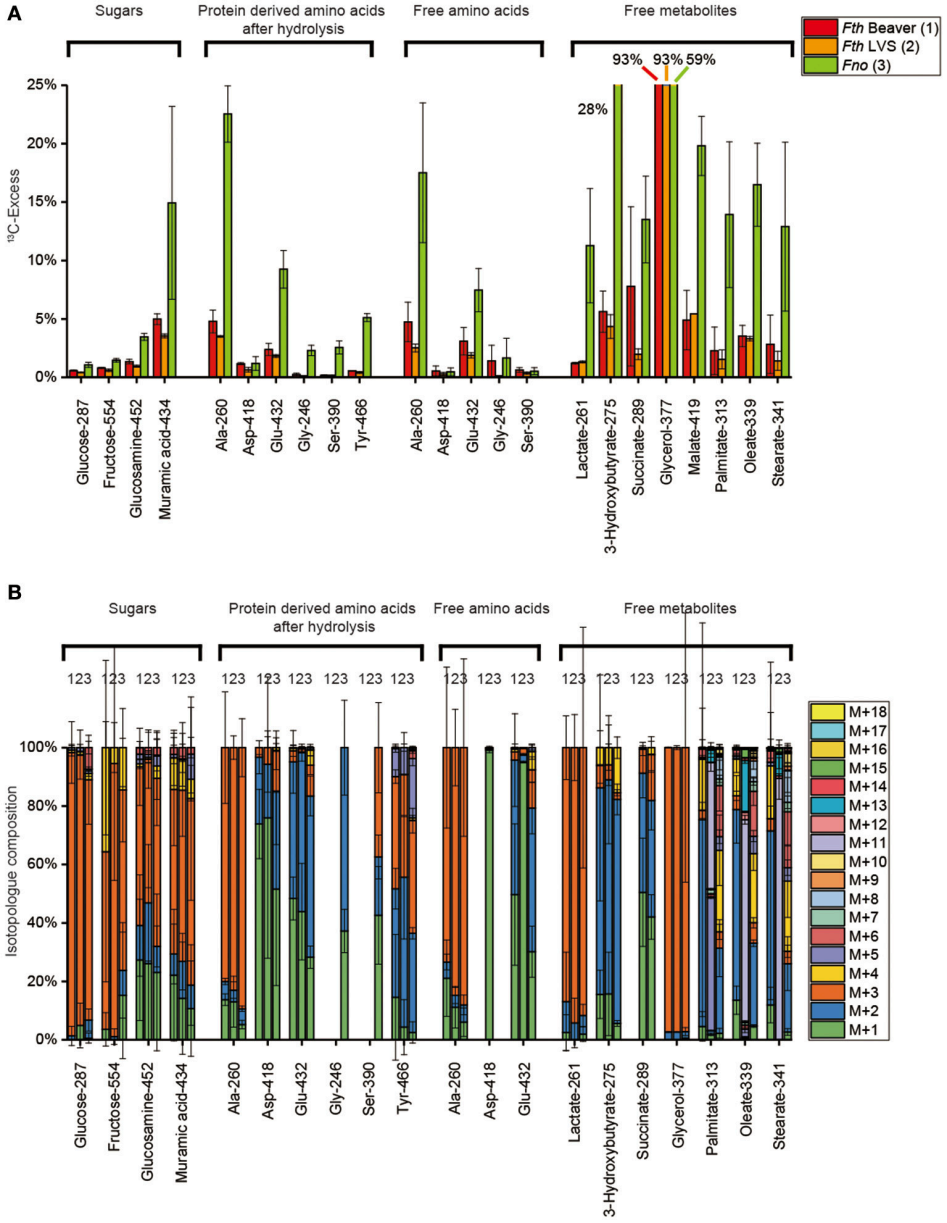
**(A)** ^13^C-Excess (mol%) and **(B)** the fractional isotopolog distributions (%) in key metabolites of *Fth* Beaver (1), *Fth* LVS (2), or *Fno* (3) grown in medium T supplied with 25 mM [U-^13^C_3_]glycerol. For numerical values, see Supplemental Tables S4, S8, S12, S16, S20, S24. For more details, see also legend to Figure 2.

Using 25 mM [U-^13^C_3_]glycerol as a substrate, GC/MS analysis suggests efficient incorporation into the cell on the basis of 93% and 59% ^13^C excess in glycerol (Figure 5A) (mainly as M+3 isotopologs, Figure 5B) from the methanolic cell extracts of the *Fth* strains and *Fno*, respectively. Conversion of labeled glycerol into [U-^13^C_3_]pyruvate could be demonstrated by ^13^C-incorporation into Ala (protein-bound and in the free form) mainly as M+3 isotopologs. Carbon flux *via* [U-^13^C_2_]-acetyl-CoA into the TCA and fatty acid biosynthesis is also clearly reflected by the M+2 isotopologs of Glu (derived from α-ketoglutarate), Asp (derived from oxaloacetate), succinate, malate, fatty acids, and 3-hydroxybutyrate. Generally, the ^13^C-enrichment values of these metabolites were much higher in *Fno* (by a factor of 2–5) in comparison to *Fth*. This is a clear indication for the better usage of glycerol by the less pathogenic *Francisella* strain *Fno*. The same holds true for products derived *via* glucogenesis and the PPP. In *Fno*, significant incorporation was found in Tyr (mainly as M+3 and M+2 from either PEP or the erythrose 4-phosphate precursor, respectively), in Ser and Gly (*via* the glucogenic intermediate, 3-phosphoglycerate), in glucosamine and muramic acid and, at lower rates, in free fructose and glucose from acid-labile polysaccharides. Again, the M+3 isotopologs were the most prominent ones, but M+1 and M+2 also made substantial fractions in the amino sugars (Figure 5B) again indicating metabolic turnover during the amino sugar biosynthesis using glycerol as a precursor. In summary, the results demonstrate that glycerol is used for gluconeogenesis quite efficiently in *Fno*, but only at minor rates in *Fth*.

### Differential substrate usage

Since medium T (see also Supplemental Tables S25, S26) contains poorly defined components such as bacto trytone (10 g/L), casamino acids (10 g/L), and brain heart infusion broth (10 g/L), it was necessary to experimentally determine the amounts of glucose, serine and glycerol in this medium. These values were important in order to normalize the incorporation rates described above for the respective ^13^C-tracers. For this purpose, medium T (without the ^13^C-tracers) was prepared following the same protocol described for the labeling experiments (with the exception of not adding the ^13^C-labeled substrates) and autoclaved. After lyophilization, a fraction was silylated as described in the Materials Methods section and analyzed by GC-MS for quantifying the amounts of free glucose, glycerol, and amino acids, e.g., serine, respectively. Another fraction of the lyophilized medium was dissolved and heated in 6 M HCl in order to hydrolyze proteins and peptides present in the medium. Subsequently, the hydrolysate was lyophilized, derivatized and again analyzed by GC-MS.

Glycerol could not be detected in any of these fractions, whereas glucose was found, in free form, in amounts of 75.3 mM. Free serine was present at a concentration of 0.5 mM, after total hydrolysis of medium T, the concentration raised to 3.1 mM. On this basis, in the labeling experiments the total concentration of glycerol was 25 mM (with 100% [U-^13^C_3_]glycerol), glucose was present at a total concentration of 94.4 mM (with 11.8% [U-^13^C_6_]glucose), and the overall concentration of serine (free form and in peptides) was 3.4 mM (with 84.3% [U-^13^C_3_]serine) (Supplemental Tables S25, S26).

**Figure 6:**
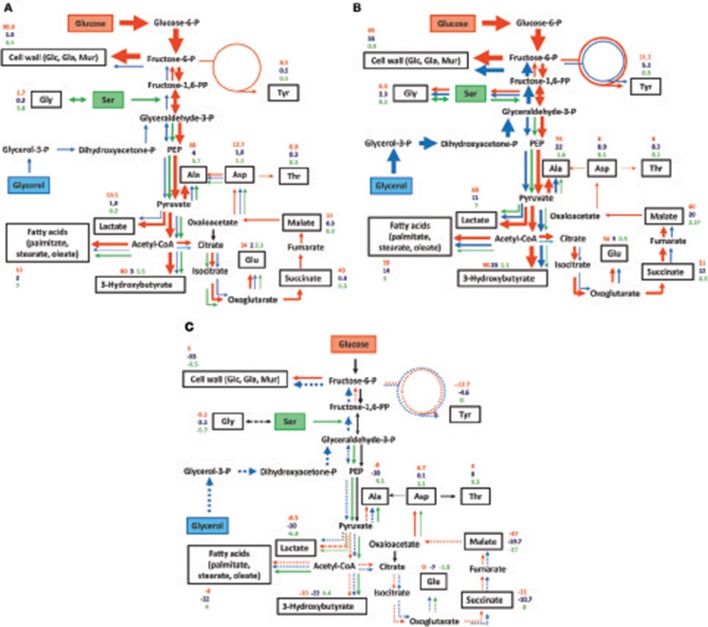
Observed metabolic pathways and fluxes in **(A)** *Fth* Beaver/*Fth* LVS, and **(B)** *Fno* from exogenous glucose (red arrows), serine (green arrows), and glycerol (blue arrows). **(C)** Differences in the metabolic fluxes between *Fth* and *Fno* (*Fth*–*Fno*). Metabolites studied by GC-MS-based isotopolog profiling are indicated by black boxes. The numbers indicate normalized overall ^13^C-enrichments (from labeled glucose, glycerol and serine in red, blue and green, respectively). The arrow widths roughly indicate the relative fluxes. Fluxes conducive to enrichments <1% are not shown. In **(C)** the numbers indicate the differences in the normalized overall ^13^C-enrichments (*Fth*–*Fno*). Higher fluxes in *Fth* are indicated by normal arrows, lower fluxes by dashed arrows.

On the basis of these findings, the ^13^C-enrichment values were normalized. For the ^13^C-glucose experiment, the measured ^13^C-enrichments values were multiplied by a factor of 8.5, for the ^13^C-serine experiment by a factor of 1.15, and for the ^13^C-glycerol experiment by a factor of 1. The normalized values are shown in Figures 6A,B for *Fth* and *Fno*, respectively. The relative fluxes are also indicated in Figures 6A,B by the arrow widths. It is immediately obvious that the cell wall sugars of *Fth* and *Fno* were predominantly (>85%) derived from the glucose supply present in medium T. Major fluxes were also observed from glucose to alanine and fatty acids (>50%). In *Fth* and *Fno*, exogenous serine (in free form or from the peptides present in medium T) was incorporated into the metabolic network only at lower rates (<10%). Similar low fluxes (<5%) were found from exogenous glycerol into the network of *Fth*. In *Fno*, however, glycerol contributed to biosynthesis at higher rates (up to 55% in cell wall sugars).

Differences in the metabolic fluxes are displayed in Figure 6C. The numbers indicate the differences between the normalized ^13^C-enrichments in *Fth* and *Fno* (*Fth*–*Fno*). Higher fluxes in *Fth* are indicated by normal arrows, whereas lower fluxes are shown by dashed arrows. It becomes evident that *Fth* is characterized by a slightly higher flux of glucose into the cell wall, but reduced fluxes (with the exception of Asp for unknown reasons) into any other metabolite under study. Moreover, higher fluxes were gleaned from serine into the downstream pathways of *Fth* (i.e., the lower part of EMP, formation of acetyl-CoA and fatty acids), but not into cell wall sugars. This points at a more bipartite metabolic network in *Fth* (i.e., with glucose feeding directly the formation of polysaccharides, and serine adding more carbon for pyruvate and actyl-CoA metabolism) in comparison to the less human-pathogenic *Fno*. Glycerol was a generally less efficient substrate in *Fth* than in *Fno*. This was especially true for cell wall sugars indicating that glucogenesis from glycerol is not a major process in *Fth* under the experimental conditions.

## Discussion

Pathogenic intracellular bacteria of the family *Francisella* can grow in the cytosolic compartments of different cell types (e.g., macrophages) in a high variety of host organisms (Santic et al., 2006; Sjostedt, 2006; Keim et al., 2007). It can be assumed that these different environments provide versatile and changing nutrient supplies. This host plasticity points at a high degree of metabolic robustness and adaptation capacities of the bacteria. The highly conserved genomes of *Francisella* strains reflect the potential usage of amino acids, glucose and glucogenetic substrates (e.g., glycerol or pyruvate) as major carbon substrates feeding a highly interconnected metabolic network. Earlier studies have indeed confirmed the role of amino acids, glucose, and glycerol as nutrients (Checroun et al., 2006; Meibom and Charbit, 2010; Santic and Abu Kwaik, 2013; Barel et al., 2015). On the other hand, the metabolic network structure of *Fth* and *Fno* could in principle allow the usage of a single carbon substrate to satisfy all metabolic demands (i.e., as a source for the generation of energy and biomass). Since multiple substrate usages have been shown in other intracellular bacterial pathogens characterized by similar metabolic capacities, it was therefore in order to analyze metabolite fluxes from the carbon sources glucose, serine, and glycerol. To this aim, we used isotopolog profiling experiments with three *Francisella* strains, i.e., the highly pathogenic *F. tularensis* subsp. *holarctica* wild type (isolated from a beaver deceased from tularemia), the attenuated live vaccine *F. tularensis* subsp. *holarctica* strain LVS, or the for humans less pathogenic *F. novicida* strain U112. As a benefit of the ^13^C-method, metabolic pathways and fluxes can be rather directly determined in contrast to transcriptomic, proteomic or metabolomic methods. Comparing the labeling profiles between the strains under study, the method should also be conducive to correlate metabolism with pathogenicity.

### Glucose

From the analysis of genome data, it was speculated that gluconeogenesis and not glycolysis is important for intracellular replication, because no phosphofructokinase was annotated within the genomes of *Ftt, Fth*, and *Fno* until recently, and genes of gluconeogenesis/glycolysis pathways have been shown to be expressed and important for intracellular growth (Meibom and Charbit, 2010; Raghunathan et al., 2010). Mutant strains of *glpX* encoding the fructose-1,6-bisphosphatase, were shown to be hampered in growth under *in vitro* and *in vivo* conditions (Kadzhaev et al., 2009; Brissac et al., 2015). On the other hand, there are general differences between the human pathogenic *Ftt* and *Fth* species and the mice pathogenic *Fno* species in carbohydrate metabolism (for review, see Kingry and Petersen, 2014). More specifically, *Fth* strains seem to metabolize glucose, but not glucose-6-phosphate *in vitro*. Only *F. hispaniensis* and *F. philomiragia* were shown to be able to metabolize glucose-1-phosphate and glucose-6-phosphate (Gyuranecz et al., 2010). Only in 2012, a gene encoding a phosphofructokinase (FTN_1210) was identified in *Fno* (Enstrom et al., 2012) providing evidence for the role of glycolysis at least in *Fno*, also confirmed by elegant labeling studies (Brissac et al., 2015).

We could now demonstrate by isotopolog profiling that exogenous glucose is by far the most efficient carbon substrate not only in *Fno*, but also in *Fth* when growing in the complex medium T. There were no significant differences between the *Fth* LVS and the *Fth* wild-type strain indicating that the reduced virulence of *Fth* LVS is not (strongly) related to glucose usage. However, as pointed out above, the direct transfer of exogenous glucose into cell wall sugars is slightly higher in *Fth* than in *Fno* (Figure 6C). The pathways of glucose degradation follow the same routes in *Fth* and *Fno*, i.e., *via* the EMP, but not the ED or PPP. Carbon flux from glucose to amino acids *via* pyruvate and intermediates of the TCA is lower in *Fth* than in *Fno* with the exception of Asp (Figure 6C). This could indicate that *Fth* utilizes additional carbon substrates for the energy generating sections of metabolism (see below) following the concepts of a bipartite metabolism in *L. pneumophila* (Gillmaier et al., 2016; Häuslein et al., 2016) and *Chlamydia trachomatis* (Mehlitz et al., 2017). Although not annotated within the genome, we could provide evidence for the presence of a putative Ala dehydrogenase or Glu/Pyr transaminase. Nevertheless, the results also demonstrate the expected activity of an aspartate 4-decarboxylase (see Figure 1).

### Glycerol

It was recently shown that glycerol is metabolized by *Fno*, mainly for gluconeogenesis during intracellular replication (Kadzhaev et al., 2009; Brissac et al., 2015). Indeed, *glpD* (glycerol-3-P dehydrogenase) is necessary for the utilization of glycerol in *Fno* (Brissac et al., 2015).

Our results confirmed these and earlier data (Petersen and Schriefer, 2005; Marinov et al., 2009; Gyuranecz et al., 2010; Huber et al., 2010), but also provided additional insights into details of species-specific differences in glycerol metabolism. As expected, carbon flux from glycerol was much higher in *Fno* than in *Fth*, and glycerol was efficiently used for gluconeogenesis, also in the presence of (unlabeled) glucose in the medium T (Figure 6C).

^13^C-Excess values of 4–5% in muramic acid and Ala from *Fth* grown in the presence of [U-^13^C_3_]glycerol clearly demonstrated that glycerol was also metabolized by *Fth*. This is noteworthy since *Fth* is known to be negative for the fermentation and metabolism of glycerol (Biolog system), but able to metabolize glycerol phosphate (Petersen and Schriefer, 2005; Gyuranecz et al., 2010; Huber et al., 2010) probably due to the presence of a glycerol phosphate transporter GlpT (see also Supplemental Figure S1) which is also necessary for the uptake of fosmidomycin (Mackie et al., 2012). However, there is also a *glpF* gene annotated in the genomes of *Francisella*, coding for an aquaglyceroporin involved in glycerol and water transport (Raghunathan et al., 2010). Moreover, three strains of *Fth* were reported to be able to produce acid from glycerol as a substrate (Marinov et al., 2009). On this basis, it is not surprising that *Fth* is using glycerol, rather it remains unclear, why *Fth* strains are only less able to utilize glycerol, also taking into account that all genes for the metabolism of glycerol are present in the genomes of *Fth* (see Supplemental Figure S1). In *F. tularensis* subsp. *tularensis* (*Ftt*), *gfpK* and *glpD* are separated by an IS-Ftu1 element, as well as in *Fth* LVS, but there, the operon is also disconnected spatially. Nevertheless, in all strains analyzed in this study, the genes for glycerol uptake and usage are present and seem to encode putative functional enzymes. Further experiments are therefore necessary to elucidate the reason for the low rate of glycerol utilization in *Fth*.

### Serine

Amino acids (Ser) are well-known to be important for intracellular multiplying *Francisella* (Meibom and Charbit, 2010; Raghunathan et al., 2010; Barel et al., 2012, 2015; Steele et al., 2013; Brown et al., 2014; Ramond et al., 2014; Gesbert et al., 2015). This was strongly supported by a report showing that the SLC family of host amino acid transporters is important for intracellular replication of *Fth* strain LVS (Barel et al., 2012). Interestingly, although *Fth* replicates in the cytosol, a similar finding of the usage of amino acids during intracellular growth and the involvement of SLC proteins of the host cell was found for *L. pneumophila*, a human pathogenic bacterium which replicates within a vacuole in alveolar macrophages (Wieland et al., 2005; Eisenreich and Heuner, 2016).

Under the *in vitro* growth conditions used in our experiments, there was only a very low flux from ^13^C-Ser into the downstream metabolites in *Fth* and especially in *Fno*, as well as a very low (if at all) flux *via* gluconeogenesis in both species (as gleaned e.g., from the apparently unlabeled cell wall-derived carbohydrates). This also came as a surprise, since gluconeogenesis is present under the *in vitro* growth conditions used here as gleaned from the glycerol experiments (Figures 6A,B).

Carbon flux from Ser to some protein-derived amino acids (e.g., Ala and Asp) was higher in *Fth* than in *Fno*. The carbon flux from Ser to Gly, however, was much higher in *Fno* than in *Fth*. For *Fth*, it was shown that the glycine cleavage system (GCS) is important for replication in Ser-limiting environments and that a *gcvT* mutant strain is auxotrophic for Ser, indicating the importance of the Ser to Gly (and *vice versa*) converting systems (Meibom and Charbit, 2010; Brown et al., 2014). Glycine-dependent Ser production in *E. coli* is only apparent when the Ser biosynthesis pathway (*serABC*) is disrupted (Ravnikar and Somerville, 1987). In addition, the GCS is the only source of Ser biosynthesis in *Fth*, but surprisingly also in *Ftt* containing a complete *serABC* pathway (Brown et al., 2014). In *Fth*, the Ser synthesis pathway (from 3-PG) is incomplete, due to a pseudo *serB* gene (P-serine phosphatase [*serB*]). This is in agreement with our results of no ^13^C flux to Ser using glucose or glycerol as a substrate.

We could now demonstrate that this is not true for *Fno*, since we obtained carbon flux from glucose and glycerol to Ser. Isotopolog profiles of protein derived Ser using ^13^C-Ser as a substrate indicated synthesis of Ser from Gly (i.e., displaying M+1 and M+2 fractions), but this was not true for free Ser collected at the stationary phase (Figure 4B). In addition, the free amino acids Ala, Ser, and Gly, as well as lactate, acquired much more ^13^C-label from Ser in *Fno*. In *Fth*, label from serine was more effectively transferred into most amino acids from the protein fraction. This may indicate that amino acids (Ser) are more efficiently used in the replicative phase of growth of *Fth*, but less in stationary phase of growth. Similar results were obtained recently for *L. pneumophila* (Gillmaier et al., 2016).

This could point again at a shift to a bipartite metabolism in *Fth* as in *L. pneumophila*, using amino acids as carbon and energy source in the exponential phase and more glucose during the post-exponential phase of growth (Eisenreich and Heuner, 2016; Gillmaier et al., 2016; Häuslein et al., 2016). In this context, it is noteworthy that species-specific differences in the metabolism of glucose were also found for different *Legionella* species (Brzuszkiewicz et al., 2013) in analogy to *Francisella* strains (Gyuranecz et al., 2010; Huber et al., 2010).

In conclusion, our findings are in agreement with multiple substrate usage by *Francisella* and species-dependent carbon fluxes from exogenous glucose, serine and glycerol. It is tempting to speculate whether the detected metabolic differences between *Fth* and *Fno* are also related to host-specific virulence, and whether the concept of metabolic virulence is a key feature for the pathogenicity of *Francisella*. Further metabolic analyses of *Francisella* strains during replication in different host cells could finally elucidate the different nutrition strategies of *Francisella* strains.

## Author contributions

KH, WE, and RG designed the study and provided facility and equipment. FC, KR, EK, IH, ES, XW, and KM performed the experiments. KH and WE wrote the paper.

### Conflict of interest statement

The authors declare that the research was conducted in the absence of any commercial or financial relationships that could be construed as a potential conflict of interest.
