# Supplementary material for: Differential Substrate Usage and Metabolic Fluxes in Francisella tularensis Subspecies holarctica and Francisella novicida
Source: Front Cell Infect Microbiol. 2017 Jun 21;7:275. doi: 10.3389/fcimb.2017.00275 (PMC5478678; doi:10.3389/fcimb.2017.00275)
Supplement: Supplementary file 1 [file Image1.PDF]

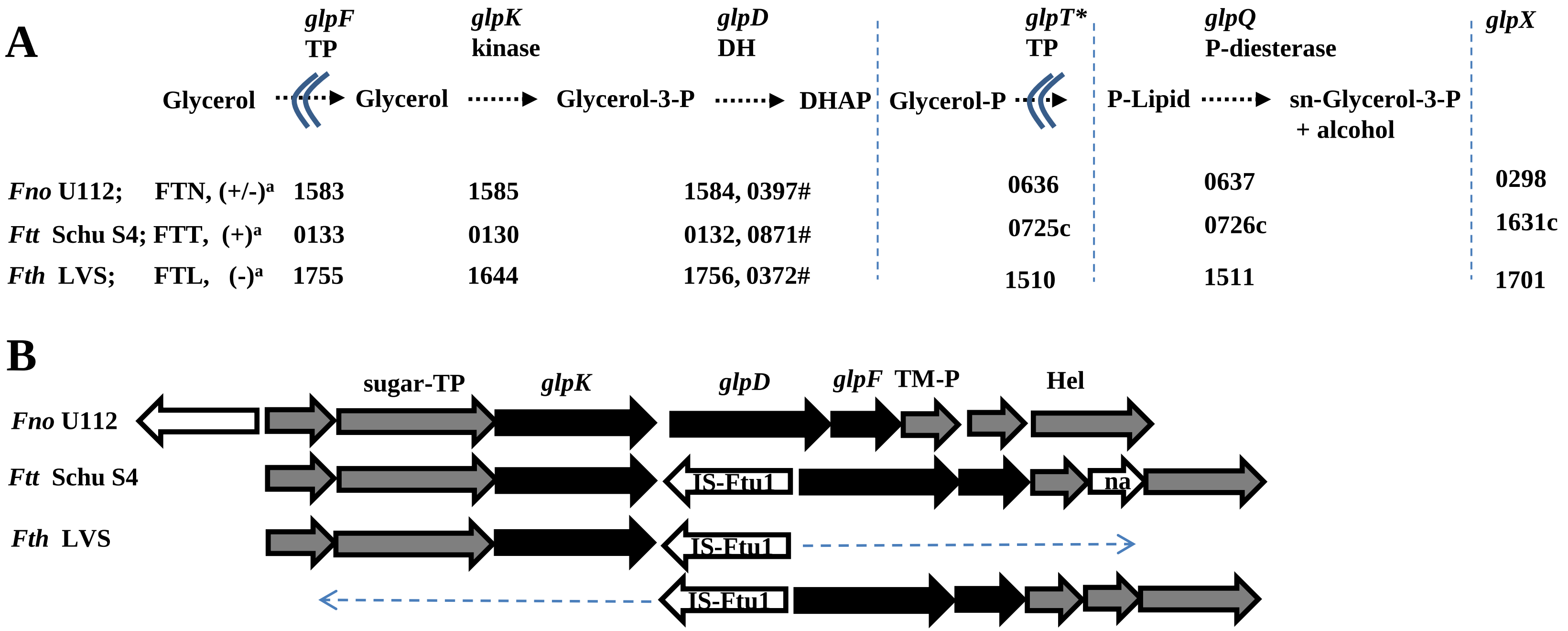

FIGURE S1: Genes involved in the utilization of glycerol in *Francisella*. Gene numbers (FTN_XXX [*Fno*], FTT_XXX [*Ftt*] and FTL_XXX [*Fth*] or names of genes are given above the arrows (A) and the respective operon structure is given (B). DH, dehydrogenase; Hel, putative helicase; na, present, but not annotated; TM-P, transmembrane protein; TP, transport protein;
#, Glycerol-3-P DH-NADP-dependent; *, also needed for the uptake of fosmidomycin (antimicrobial drug);
[a], glycerol utilization (http://www.who.int/csr/resources/publications/deliberate/WHO_CDS_EPR_2007_7/en/)
