# Supplementary material for: Differential Substrate Usage and Metabolic Fluxes in Francisella tularensis Subspecies holarctica and Francisella novicida
Source: Front Cell Infect Microbiol. 2017 Jun 21;7:275. doi: 10.3389/fcimb.2017.00275 (PMC5478678; doi:10.3389/fcimb.2017.00275)
Supplement: Supplementary file 2 [file Table1.PDF]

Supplemental Table S1 ^{13}C-Excess (mol%) of protein-bound amino acids from [U-$^{13}C_6$]glucose

^{13}C-Excess (mol%) of protein-bound amino acids from experiments with *Fth* WT, *Fth* LVS and *Fno* grown in medium T supplemented with 11 mM [U-$^{13}C_6$]glucose. Mean and SD from three independent experiments are shown.

11 mM [U-$^{13}C_6$]glucose	*Fth* WT	*Fth* LVS	*Fno*
Ala	7.82% ± 0.37%	7.87% ± 0.59%	9.05% ± 0.79%
Asp	1.68% ± 0.33%	1.50% ± 0.64%	0.72% ± 0.32%
Glu	3.91% ± 0.24%	3.82% ± 0.22%	4.15% ± 0.65%
Gly	0.35% ± 0.13%	0.14% ± 0.12%	0.83% ± 0.19%
His	0.14% ± 0.06%	0.10% ± 0.12%	0.06% ± 0.03%
Ile	0.09% ± 0.02%	0.08% ± 0.05%	0.08% ± 0.05%
Leu	0.00% ± 0.00%	0.01% ± 0.01%	0.01% ± 0.01%
Lys	0.05% ± 0.01%	0.09% ± 0.02%	0.07% ± 0.03%
Phe	0.30% ± 0.02%	0.13% ± 0.22%	0.06% ± 0.12%
Pro	0.15% ± 0.01%	0.30% ± 0.11%	0.14% ± 0.04%
Met		0.49% ± 0.26%	0.50% ± 0.31%
Ser	0.17% ± 0.03%	0.13% ± 0.05%	0.87% ± 0.24%
Thr	0.63% ± 0.02%	0.58% ± 0.06%	0.66% ± 0.12%
Tyr	1.13% ± 0.07%	0.77% ± 0.07%	2.48% ± 0.29%
Val	0.21% ± 0.01%	0.05% ± 0.07%	0.12% ± 0.10%

Supplemental Table S2 ^{13}C-Excess (mol%) of protein-bound amino acids from [1,2-$^{13}C_2$]glucose

^{13}C-Excess (mol%) of protein-bound amino acids from experiments with *Fth* WT, *Fth* LVS and *Fno* grown in medium T supplemented with 11 mM [1,2-$^{13}C_2$]glucose. Mean and SD from two independent experiments are shown.

11 mM [1,2-$^{13}C_2$]glucose	*Fth* WT	*Fth* LVS	*Fno*
Ala	2.62% ± 0.09%	3.44% ± 0.12%	3.63% ± 0.07%
Asp	0.57% ± 0.00%	1.02% ± 0.27%	0.47% ± 0.09%
Glu	1.62% ± 0.10%	2.37% ± 0.14%	2.54% ± 0.03%
Gly	0.31% ± 0.03%	0.02% ± 0.01%	0.20% ± 0.02%
His	0.14% ± 0.08%	0.15% ± 0.00%	
Ile	0.12% ± 0.00%	0.04% ± 0.01%	0.12% ± 0.00%
Leu	0.00% ± 0.00%	0.03% ± 0.00%	0.07% ± 0.00%
Lys	0.08% ± 0.01%	0.20% ± 0.01%	
Phe	0.23% ± 0.00%	0.16% ± 0.04%	0.34% ± 0.05%
Pro	1.25% ± 0.46%	0.20% ± 0.07%	0.81% ± 0.11%
Met	0.18% ± 0.01%		
Ser	0.20% ± 0.01%	0.38% ± 0.01%	1.28% ± 0.07%
Thr	0.72% ± 0.02%	0.13% ± 0.03%	0.44% ± 0.13%
Tyr	0.53% ± 0.09%	0.65% ± 0.43%	1.72% ± 0.05%
Val	0.17% ± 0.04%	0.02% ± 0.01%	0.09% ± 0.01%

Supplemental Table S3 ^{13}C-Excess (mol%) of protein-bound amino acids from [U-$^{13}C_3$]serine

^{13}C-Excess (mol%) of protein-bound amino acids from experiments with *Fth* WT, *Fth* LVS and *Fno* grown in medium T supplemented with 3 mM [U-$^{13}C_3$]serine. Mean and SD from three independent experiments are shown.

3 mM [U-$^{13}C_3$]serine	*Fth* WT	*Fth* LVS	*Fno*
Ala	5.13% ± 0.06%	5.79% ± 0.12%	1.44% ± 0.04%
Asp	0.99% ± 0.03%	1.20% ± 0.08%	0.14% ± 0.08%
Glu	2.09% ± 0.02%	2.62% ± 0.02%	0.37% ± 0.04%
Gly	3.68% ± 0.25%	2.68% ± 0.27%	8.28% ± 0.64%
His	0.07% ± 0.01%	0.04% ± 0.01%	0.05% ± 0.00%
Ile	0.07% ± 0.00%	0.11% ± 0.03%	0.10% ± 0.01%
Leu	0.01% ± 0.01%	0.03% ± 0.03%	0.00% ± 0.00%
Lys	0.10% ± 0.02%	0.09% ± 0.01%	0.09% ± 0.01%
Phe	3.32% ± 0.52%	0.55% ± 0.09%	0.17% ± 0.06%
Pro	0.11% ± 0.03%	0.07% ± 0.00%	0.18% ± 0.01%
Met	0.21% ± 0.02%	0.20% ± 0.02%	0.25% ± 0.09%
Ser	12.92% ± 0.12%	12.39% ± 0.20%	16.02% ± 0.57%
Thr	0.53% ± 0.04%	0.58% ± 0.08%	0.51% ± 0.12%
Tyr	0.62% ± 0.55%	0.16% ± 0.09%	0.38% ± 0.28%
Val	0.05% ± 0.01%	0.06% ± 0.06%	0.06% ± 0.04%

3 mM [U-$^{13}C_3$]serine	*Fth* WT and *Fth* LVS	*Fno*
Ser	12.66% ± 0.37%	16.02% ± 0.57%

Supplemental Table S4 ^{13}C-Excess (mol%) of protein-bound amino acids from [U-$^{13}C_3$] glycerol

^{13}C-Excess (mol%) of protein-bound amino acids from experiments with *Fth* WT, *Fth* LVS and *Fno* grown in medium T supplemented with 25 mM [U-$^{13}C_3$] glycerol. Mean and SD from three independent experiments are shown.

25 mM [U-$^{13}C_3$]glycerol	*Fth* WT	*Fth* LVS	*Fno*
Ala	4.79% ± 0.97%	3.51% ± 0.08%	22.54% ± 2.41%
Asp	1.17% ± 0.09%	0.65% ± 0.20%	1.19% ± 0.58%
Glu	2.39% ± 0.51%	1.82% ± 0.13%	9.25% ± 1.61%
Gly	0.25% ± 0.11%	0.14% ± 0.01%	2.30% ± 0.45%
His	0.08% ± 0.02%	0.08% ± 0.00%	0.13% ± 0.12%
Ile	0.09% ± 0.03%	0.07% ± 0.04%	0.11% ± 0.02%
Leu	0.05% ± 0.04%	0.06% ± 0.03%	0.03% ± 0.02%
Lys	0.09% ± 0.03%	0.14% ± 0.01%	0.09% ± 0.04%
Phe	0.62% ± 0.41%	0.34% ± 0.34%	0.18% ± 0.14%
Pro	0.17% ± 0.06%	0.33% ± 0.03%	0.20% ± 0.11%
Met	0.33% ± 0.00%	0.29% ± 0.01%	0.25% ± 0.03%
Ser	0.17% ± 0.05%	0.16% ± 0.03%	2.56% ± 0.56%
Thr	0.46% ± 0.08%	0.22% ± 0.13%	0.49% ± 0.09%
Tyr	0.55% ± 0.02%	0.41% ± 0.08%	5.12% ± 0.33%
Val	0.03% ± 0.00%	0.04% ± 0.02%	0.24% ± 0.02%

Supplemental Table S5 ^{13}C-Excess (mol%) of polar metabolites from [U-$^{13}C_6$]glucose

^{13}C-Excess (mol%) of polar metabolites from experiments with *Fth* WT, *Fth* LVS and *Fno* grown in medium T supplemented with 11 mM [U-$^{13}C_6$]glucose. Mean and SD from three independent experiments are shown.

11 mM [U-$^{13}C_6$]glucose	*Fth* WT	*Fth* LVS	*Fno*
Lactate	7.31% ± 0.78%	7.67% ± 1.39%	7.80% ± 0.70%
Glycolic acid	10.54% ± 0.46%	9.39% ± 1.30%	9.40% ± 1.65%
Alanine	9.96% ± 0.45%	9.89% ± 0.11%	10.00% ± 0.82%
Glycine	0.27% ± 0.03%	0.20% ± 0.22%	0.67% ± 0.27%
Oxalic acid	2.61% ± 0.18%	2.04% ± 0.21%	1.54% ± 0.43%
3-Hydroxy butyric acid	9.71% ± 0.42%	10.14% ± 0.26%	11.94% ± 0.70%
Valine	0.18% ± 0.02%	0.06% ± 0.06%	0.07% ± 0.07%
leucine	0.03% ± 0.00%	0.02% ± 0.01%	0.01% ± 0.01%
Isoleucine	0.03% ± 0.01%	0.03% ± 0.02%	0.05% ± 0.02%
Succinic acid	3.78% ± 0.33%	5.70% ± 0.91%	6.36% ± 1.16%
Fumaric acid	1.09% ± 0.78%	1.65% ± 0.29%	2.07% ± 0.72%
Glycerol	8.15% ± 0.49%	7.64% ± 0.84%	9.29% ± 1.25%
Methionine	0.12% ± 0.03%	0.08% ± 0.06%	0.08% ± 0.03%
Serine	0.53% ± 0.04%	0.45% ± 0.04%	0.56% ± 0.06%
Threonine	0.20% ± 0.02%	0.16% ± 0.09%	0.19% ± 0.06%
Phenylalanine	1.14% ± 0.10%	2.27% ± 0.30%	0.20% ± 0.05%
Malic acid	2.81% ± 0.20%	2.95% ± 0.31%	7.65% ± 1.75%
Aspartate	0.41% ± 0.02%	0.36% ± 0.20%	0.09% ± 0.05%
Glutamate	6.21% ± 0.27%	4.61% ± 0.63%	3.48% ± 0.33%
Palmitic acid	6.50% ± 0.47%	5.43% ± 1.48%	6.25% ± 1.03%
Oleic acid	6.92% ± 0.67%	6.77% ± 0.40%	7.50% ± 0.47%
Stearic acid	8.03% ± 1.46%	7.22% ± 1.50%	8.06% ± 0.77%
Citric acid	0.16% ± 0.09%	0.35% ± 0.08%	0.68% ± 0.44%

Supplemental Table S6 ^{13}C-Excess (mol%) of polar metabolites from [1,2-$^{13}C_2$]glucose

^{13}C-Excess (mol%) of polar metabolites from experiments with *Fth* WT grown in medium T supplemented with 11 mM [1,2-$^{13}C_2$]glucose. Mean and SD from two independent experiments are shown.

11 mM [1,2-$^{13}C_2$]glucose	*Fth* WT
Lactate	5.04% ± 0.38%
Glycolic acid	4.87% ± 0.12%
Alanine	3.16% ± 0.23%
Glycine	0.13% ± 0.03%
Oxalic acid	0.01% ± 0.01%
3-Hydroxy butyric acid	3.51% ± 0.12%
Valine	0.16% ± 0.00%
leucine	0.03% ± 0.00%
Isoleucine	0.04% ± 0.00%
Succinic acid	0.53% ± 0.04%
Fumaric acid	0.24% ± 0.01%
Glycerol	2.74% ± 0.23%
Methionine	0.11% ± 0.01%
Serine	0.43% ± 0.04%
Threonine	0.18% ± 0.01%
Phenylalanine	0.48% ± 0.04%
Malic acid	1.24% ± 0.02%
Aspartate	0.19% ± 0.02%
Glutamate	2.04% ± 0.17%
Palmitic acid	2.90% ± 0.17%
Oleic acid	3.44% ± 0.26%
Stearic acid	3.98% ± 0.21%

Supplemental Table S7 ^{13}C-Excess (mol%) of polar metabolites from [U-$^{13}C_3$]serine

^{13}C-Excess (mol%) of polar metabolites from experiments with *Fth* WT, *Fth* LVS and *Fno* grown in medium T supplemented with 3 mM [U-$^{13}C_3$]serine. Mean and SD from three independent experiments are shown.

3 mM [U-$^{13}C_3$]serine	*Fth* WT	*Fth* LVS	*Fno*
Lactate	0.12% ± 0.01%	0.12% ± 0.01%	5.70% ± 0.44%
Glycolic acid	0.41% ± 0.17%	0.31% ± 0.12%	0.06% ± 0.06%
Alanine	0.85% ± 0.03%	0.96% ± 0.08%	5.73% ± 0.38%
Glycine	1.67% ± 0.01%	1.27% ± 0.02%	8.48% ± 0.13%
Oxalic acid	0.00% ± 0.00%	0.00% ± 0.00%	0.00% ± 0.00%
3-Hydroxy butyric acid	3.02% ± 0.17%	2.40% ± 0.09%	0.85% ± 0.07%
Valine	0.09% ± 0.03%	0.08% ± 0.04%	0.09% ± 0.03%
leucine	0.05% ± 0.01%	0.05% ± 0.01%	0.06% ± 0.02%
Isoleucine	0.14% ± 0.04%	0.15% ± 0.04%	0.14% ± 0.05%
Succinic acid	0.21% ± 0.03%	0.23% ± 0.07%	0.21% ± 0.00%
Fumaric acid	0.18% ± 0.06%	0.28% ± 0.26%	0.36% ± 0.15%
Glycerol	0.26% ± 0.22%	0.27% ± 0.21%	0.34% ± 0.16%
Methionine	0.22% ± 0.08%	0.16% ± 0.04%	0.12% ± 0.01%
Serine	9.58% ± 0.88%	5.71% ± 0.08%	20.38% ± 0.60%
Threonine	0.14% ± 0.08%	0.17% ± 0.00%	0.19% ± 0.11%
Phenylalanine	0.17% ± 0.04%	0.13% ± 0.01%	0.28% ± 0.11%
Malic acid	0.92% ± 0.24%	2.10% ± 0.93%	2.56% ± 0.18%
Aspartate	0.11% ± 0.05%	0.08% ± 0.08%	0.14% ± 0.06%
Glutamate	0.51% ± 0.05%	0.39% ± 0.00%	0.27% ± 0.01%
Palmitic acid	5.05% ± 0.54%	5.30% ± 0.36%	0.88% ± 0.31%
Oleic acid	8.97% ± 1.02%	8.66% ± 0.49%	3.75% ± 0.99%
Stearic acid	2.86% ± 0.08%	2.69% ± 0.12%	1.29% ± 0.55%
Citric acid	2.91% ± 0.00%	0.12% ± 0.01%	0.38% ± 0.11%

Supplemental Table S8 ^{13}C-Excess (mol%) of polar metabolites from [U-$^{13}C_3$] glycerol

^{13}C-Excess (mol%) of polar metabolites from experiments with *Fth* WT, *Fth* LVS and *Fno* grown in medium T supplemented with 25 mM [U-$^{13}C_3$] glycerol. Mean and SD from three independent experiments are shown.

25 mM [U-$^{13}C_3$]glycerol	*Fth* WT	*Fth* LVS	*Fno*
Lactate	1.21% ± 0.07%	1.32% ± 0.08%	11.27% ± 4.89%
Glycolic acid	6.95% ± 4.99%	2.29% ± 0.12%	6.24% ± 3.82%
Alanine	4.74% ± 1.70%	2.52% ± 0.34%	17.51% ± 5.98%
Glycine	1.40% ± 1.35%	0.11% ± 0.05%	1.65% ± 1.71%
Oxalic acid	2.06% ± 2.06%	0.05% ± 0.05%	0.81% ± 1.11%
3-Hydroxy butyric acid	5.63% ± 1.76%	4.35% ± 1.02%	28.43% ± 3.28%
Valine	0.19% ± 0.16%	0.05% ± 0.03%	0.21% ± 0.18%
leucine	0.04% ± 0.04%	0.01% ± 0.01%	0.03% ± 0.01%
Isoleucine	0.14% ± 0.10%	0.07% ± 0.03%	0.15% ± 0.05%
Succinic acid	7.79% ± 6.82%	1.98% ± 0.46%	13.50% ± 3.71%
Fumaric acid	4.83% ± 4.32%	0.51% ± 0.38%	6.75% ± 4.12%
Glycerol	92.98% ± 0.11%	92.70% ± 0.57%	58.52% ± 26.17%
Methionine	0.18% ± 0.18%	0.10% ± 0.10%	0.11% ± 0.08%
Serine	0.63% ± 0.20%	0.36% ± 0.10%	0.53% ± 0.30%
Threonine	0.25% ± 0.06%	0.18% ± 0.12%	0.26% ± 0.09%
Phenylalanine	0.75% ± 0.10%	1.15% ± 0.05%	0.19% ± 0.05%
Malic acid	4.89% ± 2.55%	5.42% ± 0.71%	19.81% ± 2.53%
Aspartate	0.55% ± 0.43%	0.29% ± 0.13%	0.47% ± 0.34%
Glutamate	3.10% ± 1.18%	1.88% ± 0.22%	7.47% ± 1.86%
Palmitic acid	2.28% ± 2.03%	1.54% ± 0.81%	13.93% ± 6.23%
Oleic acid	3.53% ± 0.92%	3.31% ± 0.19%	16.48% ± 3.55%
Stearic acid	2.83% ± 2.50%	1.41% ± 0.82%	12.90% ± 7.23%
Citric acid	1.28% ± 0.88%	1.08% ± 0.23%	5.15% ± 5.58%

Supplemental Table S9 ^{13}C-Excess (mol%) of sugars from [U-$^{13}C_6$]glucose

^{13}C-Excess (mol%) of sugars from experiments with *Fth* WT, *Fth* LVS and *Fno* grown in medium T supplemented with 11 mM [U-$^{13}C_6$]glucose. Mean and SD from three independent experiments are shown.

11 mM [U-$^{13}C_6$]glucose	*Fth* WT	*Fth* LVS	*Fno*
Glucose in glycogen	10.41% ± 0.44%	10.60% ± 0.54%	9.68% ± 0.94%
Free fructose	12.48% ± 0.77%	11.71% ± 0.54%	12.18% ± 0.39%
Glucosamine	10.77% ± 1.23%	10.16% ± 0.87%	10.22% ± 0.95%
Muramic acid	12.19% ± 1.55%	11.92% ± 1.52%	10.05% ± 1.44%

11 mM [U-$^{13}C_6$]glucose	*Fth* WT and *Fth* LVS	*Fno*
averaged over the four hexoses	11.16% ± 0.90%	10.53% ± 1.12%

Supplemental Table S10 ^{13}C-Excess (mol%) of sugars from [1,2-$^{13}C_2$]glucose

^{13}C-Excess (mol%) of sugars from experiments with *Fth* WT grown in medium T supplemented with 11 mM [1,2-$^{13}C_2$]glucose. Mean and SD from two independent experiments are shown.

11 mM [1,2-$^{13}C_2$]glucose	*Fth* WT
Glucose in glycogen	4.55% ± 0.09%
Free fructose	4.89% ± 0.14%
Glucosamine	5.25% ± 0.01%
Muramic acid	7.85% ± 0.03%

Supplemental Table S11 ^{13}C-Excess (mol%) of sugars from [U-$^{13}C_3$]serine

^{13}C-Excess (mol%) of sugars from experiments with *Fth* WT, *Fth* LVS and *Fno* grown in medium T supplemented with 3 mM [U-$^{13}C_3$]serine. Mean and SD from three independent experiments are shown.

3 mM [U-$^{13}C_3$]serine	*Fth* WT	*Fth* LVS	*Fno*
Glucose in glycogen	0.06% ± 0.05%	0.05% ± 0.04%	0.02% ± 0.02%
Free fructose	0.55% ± 0.04%	0.53% ± 0.00%	0.46% ± 0.00%
Glucosamine	0.42% ± 0.11%	0.31% ± 0.04%	0.39% ± 0.01%
Muramic acid	0.66% ± 0.14%	0.59% ± 0.04%	1.83% ± 0.69%

Supplemental Table S12 ^{13}C-Excess (mol%) of sugars from [U-$^{13}C_3$] glycerol

^{13}C-Excess (mol%) of sugars from experiments with *Fth* WT, *Fth* LVS and *Fno* grown in medium T supplemented with 25 mM [U-$^{13}C_3$] glycerol. Mean and SD from three independent experiments are shown.

25 mM [U-$^{13}C_3$]glycerol	*Fth* WT	*Fth* LVS	*Fno*
Glucose in glycogen	0.58% ± 0.04%	0.42% ± 0.04%	1.07% ± 0.22%
Free fructose	0.80% ± 0.05%	0.59% ± 0.11%	1.46% ± 0.16%
Glucosamine	1.34% ± 0.20%	0.95% ± 0.08%	3.46% ± 0.28%
Muramic acid	4.98% ± 0.46%	3.55% ± 0.17%	14.93% ± 8.25%

Supplemental Table S13 Relative fractions of isotopologues (mol%) of amino acids from experiments with *Fth* WT, *Fth* LVS and *Fno* grown in medium T supplemented with 11 mM [U-$^{13}C_6$]glucose. M+x represents the mass of the unlabelled metabolite plus x labelled ^{13}C-atoms. Mean and SD from three independent experiments are shown.

11 mM [U-$^{13}C_6$]glucose *Fth* WT								
	Alanine	Aspartate	Glutamate	Glycine	Phenylalanine	Serine	Threonine	Tyrosine
M+1	1.80 % ± 0.30 %	2.51 % ± 0.64 %	5.63 % ± 0.45 %	0.53 % ± 0.20 %	1.17 % ± 0.37 %	0.03 % ± 0.08 %	1.78 % ± 0.33 %	0.18 % ± 0.39 %
M+2	1.33 % ± 0.06 %	1.61 % ± 0.40 %	5.60 % ± 0.32 %	0.09 % ± 0.07 %	0.01 % ± 0.02 %	0.00 % ± 0.00 %	0.37 % ± 0.13 %	0.97 % ± 0.19 %
M+3	6.34 % ± 0.35 %	0.33 % ± 0.07 %	0.61 % ± 0.08 %		0.12 % ± 0.06 %	0.16 % ± 0.04 %	0.00 % ± 0.00 %	1.00 % ± 0.09 %
M+4		0.00 % ± 0.00 %	0.18 % ± 0.04 %		0.25 % ± 0.02 %		0.00 % ± 0.00 %	0.54 % ± 0.05 %
M+5			0.03 % ± 0.00 %		0.03 % ± 0.02 %			0.26 % ± 0.03 %
M+6					0.00 % ± 0.00 %			0.11 % ± 0.03 %
M+7					0.00 % ± 0.00 %			0.00 % ± 0.01 %
M+8					0.00 % ± 0.00 %			0.01 % ± 0.01 %
M+9					0.00 % ± 0.00 %			0.08 % ± 0.05 %

11 mM [U-$^{13}C_6$]glucose *Fth* LVS								
	Alanine	Aspartate	Glutamate	Glycine	Phenylalanine	Serine	Threonine	Tyrosine
M+1	1.94 % ± 0.48 %	2.62 % ± 1.32 %	5.31 % ± 0.57 %	0.20 % ± 0.21 %	0.66 % ± 0.50 %	0.00 % ± 0.00 %	1.68 % ± 0.58 %	0.19 % ± 0.30 %
M+2	1.24 % ± 0.24 %	1.26 % ± 0.72 %	5.72 % ± 0.49 %	0.03 % ± 0.06 %	0.05 % ± 0.08 %	0.00 % ± 0.00 %	0.32 % ± 0.27 %	0.56 % ± 0.26 %
M+3	6.40 % ± 0.36 %	0.28 % ± 0.09 %	0.54 % ± 0.09 %		0.30 % ± 0.12 %	0.13 % ± 0.06 %	0.00 % ± 0.00 %	0.67 % ± 0.11 %
M+4		0.00 % ± 0.01 %	0.15 % ± 0.05 %		0.38 % ± 0.04 %		0.00 % ± 0.00 %	0.44 % ± 0.09 %
M+5			0.02 % ± 0.01 %		0.06 % ± 0.02 %			0.25 % ± 0.04 %
M+6					0.00 % ± 0.00 %			0.06 % ± 0.04 %
M+7					0.00 % ± 0.00 %			0.00 % ± 0.01 %
M+8					0.00 % ± 0.00 %			0.02 % ± 0.01 %
M+9					0.00 % ± 0.00 %			0.01 % ± 0.02 %

11 mM [U-$^{13}C_6$]glucose *Fno*								
	Alanine	Aspartate	Glutamate	Glycine	Phenylalanine	Serine	Threonine	Tyrosine
M+1	1.26 % ± 0.39 %	1.22 % ± 0.49 %	4.74 % ± 1.18 %	0.57 % ± 0.20 %	0.62 % ± 0.45 %	0.61 % ± 0.51 %	1.79 % ± 0.66 %	0.35 % ± 0.39 %
M+2	1.06 % ± 0.22 %	0.55 % ± 0.36 %	6.65 % ± 0.76 %	0.55 % ± 0.15 %	0.00 % ± 0.00 %	0.16 % ± 0.17 %	0.42 % ± 0.23 %	1.99 % ± 0.33 %
M+3	7.92 % ± 0.65 %	0.18 % ± 0.14 %	0.61 % ± 0.17 %		0.00 % ± 0.00 %	0.56 % ± 0.12 %	0.00 % ± 0.00 %	2.17 % ± 0.22 %
M+4		0.00 % ± 0.00 %	0.19 % ± 0.10 %		0.14 % ± 0.02 %		0.00 % ± 0.00 %	1.65 % ± 0.21 %
M+5			0.02 % ± 0.01 %		0.00 % ± 0.01 %			0.43 % ± 0.06 %
M+6					0.00 % ± 0.00 %			0.28 % ± 0.05 %
M+7					0.00 % ± 0.00 %			0.10 % ± 0.06 %
M+8					0.04 % ± 0.06 %			0.01 % ± 0.02 %
M+9					0.00 % ± 0.00 %			0.02 % ± 0.05 %

Supplemental Table S14 Relative fractions of isotopologues (mol%) of amino acids from experiments with *Fth* WT, *Fth* LVS and *Fno* grown in medium T supplemented with 11 mM [1,2-$^{13}C_2$]glucose. M+x represents the mass of the unlabelled metabolite plus x labelled ^{13}C-atoms. Mean and SD from three independent experiments are shown.

11 mM [1,2-$^{13}C_2$]glucose *Fth* WT								
	Alanine	Aspartate	Glutamate	Glycine	Phenylalanine	Serine	Threonine	Tyrosine
M+1	0.72 % ± 0.34 %	0.98 % ± 0.42 %	2.60 % ± 0.54 %	0.29 % ± 0.11 %	1.35 % ± 0.39 %	0.00 % ± 0.00 %	1.69 % ± 0.27 %	0.00 % ± 0.00 %
M+2	3.49 % ± 0.19 %	0.47 % ± 0.09 %	2.55 % ± 0.24 %	0.17 % ± 0.02 %	0.08 % ± 0.09 %	0.00 % ± 0.00 %	0.55 % ± 0.10 %	1.58 % ± 0.27 %
M+3	0.06 % ± 0.02 %	0.12 % ± 0.06 %	0.11 % ± 0.07 %		0.00 % ± 0.00 %	0.21 % ± 0.03 %	0.00 % ± 0.00 %	0.08 % ± 0.10 %
M+4		0.00 % ± 0.00 %	0.00 % ± 0.00 %		0.13 % ± 0.02 %		0.00 % ± 0.00 %	0.00 % ± 0.00 %
M+5			0.01 % ± 0.01 %		0.00 % ± 0.00 %			0.06 % ± 0.01 %
M+6					0.00 % ± 0.00 %			0.01 % ± 0.02 %
M+7					0.00 % ± 0.00 %			0.00 % ± 0.00 %
M+8					0.00 % ± 0.00 %			0.00 % ± 0.00 %
M+9					0.00 % ± 0.00 %			0.01 % ± 0.02 %

11 mM [1,2-$^{13}C_2$]glucose *Fth* LVS								
	Alanine	Aspartate	Glutamate	Glycine	Phenylalanine	Serine	Threonine	Tyrosine
M+1	1.54 % ± 0.09 %	1.35 % ± 0.54 %	3.51 % ± 0.24 %	0.01 % ± 0.02 %	0.00 % ± 0.00 %	0.13 % ± 0.20 %	0.00 % ± 0.00 %	0.00 % ± 0.00 %
M+2	4.33 % ± 0.17 %	1.28 % ± 0.19 %	3.78 % ± 0.32 %	0.02 % ± 0.02 %	0.42 % ± 0.11 %	0.50 % ± 0.10 %	0.00 % ± 0.01 %	1.17 % ± 0.23 %
M+3	0.04 % ± 0.02 %	0.04 % ± 0.03 %	0.17 % ± 0.08 %		0.00 % ± 0.00 %	0.00 % ± 0.00 %	0.17 % ± 0.07 %	0.14 % ± 0.14 %
M+4		0.00 % ± 0.00 %	0.06 % ± 0.01 %		0.01 % ± 0.01 %		0.00 % ± 0.00 %	0.09 % ± 0.15 %
M+5			0.00 % ± 0.00 %		0.04 % ± 0.03 %			0.07 % ± 0.12 %
M+6					0.02 % ± 0.02 %			0.08 % ± 0.10 %
M+7					0.01 % ± 0.01 %			0.04 % ± 0.09 %
M+8					0.01 % ± 0.02 %			0.10 % ± 0.14 %
M+9					0.02 % ± 0.02 %			0.08 % ± 0.16 %

11 mM [1,2-$^{13}C_2$]glucose *Fno*								
	Alanine	Aspartate	Glutamate	Glycine	Phenylalanine	Serine	Threonine	Tyrosine
M+1	0.98 % ± 0.12 %	0.32 % ± 0.16 %	3.14 % ± 0.18 %	0.38 % ± 0.11 %	0.00 % ± 0.00 %	2.71 % ± 0.31 %	0.00 % ± 0.00 %	0.62 % ± 0.59 %
M+2	4.84 % ± 0.09 %	0.71 % ± 0.12 %	4.31 % ± 0.11 %	0.00 % ± 0.01 %	0.48 % ± 0.32 %	0.56 % ± 0.16 %	0.78 % ± 0.14 %	3.23 % ± 0.36 %
M+3	0.08 % ± 0.06 %	0.02 % ± 0.03 %	0.20 % ± 0.08 %		0.00 % ± 0.00 %	0.00 % ± 0.00 %	0.00 % ± 0.00 %	0.26 % ± 0.30 %
M+4		0.02 % ± 0.02 %	0.08 % ± 0.04 %		0.02 % ± 0.03 %		0.05 % ± 0.08 %	0.00 % ± 0.00 %
M+5			0.01 % ± 0.01 %		0.09 % ± 0.11 %			0.23 % ± 0.26 %
M+6					0.02 % ± 0.02 %			0.11 % ± 0.18 %
M+7					0.08 % ± 0.13 %			0.15 % ± 0.18 %
M+8					0.08 % ± 0.09 %			0.00 % ± 0.01 %
M+9					0.04 % ± 0.08 %			0.52 % ± 0.63 %

Supplemental Table S15 Relative fractions of isotopologues (mol%) of amino acids from experiments with *Fth* WT, *Fth* LVS and *Fno* grown in medium T supplemented with 3 mM [U-$^{13}C_3$]serine. M+x represents the mass of the unlabelled metabolite plus x labelled ^{13}C-atoms. Mean and SD from three independent experiments are shown.

3 mM [U-$^{13}C_3$]serine *Fth* WT								
	Alanine	Aspartate	Glutamate	Glycine	Phenylalanine	Serine	Threonine	Tyrosine
M+1	1.07 % ± 0.32 %	2.09 % ± 0.28 %	2.95 % ± 0.26 %	0.66 % ± 0.14 %	0.47 % ± 0.35 %	0.92 % ± 0.24 %	1.60 % ± 0.32 %	0.08 % ± 0.20 %
M+2	0.64 % ± 0.06 %	0.79 % ± 0.09 %	3.38 % ± 0.17 %	3.35 % ± 0.22 %	0.00 % ± 0.00 %	0.84 % ± 0.07 %	0.27 % ± 0.15 %	0.20 % ± 0.15 %
M+3	4.34 % ± 0.06 %	0.10 % ± 0.02 %	0.13 % ± 0.09 %		0.00 % ± 0.00 %	12.06 % ± 0.11 %	0.00 % ± 0.00 %	0.01 % ± 0.02 %
M+4		0.00 % ± 0.00 %	0.05 % ± 0.03 %		0.12 % ± 0.02 %		0.00 % ± 0.00 %	0.00 % ± 0.00 %
M+5			0.03 % ± 0.01 %		0.00 % ± 0.00 %			0.08 % ± 0.03 %
M+6					0.00 % ± 0.00 %			0.00 % ± 0.00 %
M+7					0.00 % ± 0.00 %			0.00 % ± 0.00 %
M+8					0.00 % ± 0.00 %			0.00 % ± 0.00 %
M+9					0.00 % ± 0.00 %			0.52 % ± 0.58 %

3 mM [U-$^{13}C_3$]serine *Fth* LVS								
	Alanine	Aspartate	Glutamate	Glycine	Phenylalanine	Serine	Threonine	Tyrosine
M+1	0.80 % ± 0.45 %	2.05 % ± 0.37 %	3.31 % ± 0.28 %	0.50 % ± 0.18 %	0.16 % ± 0.14 %	0.15 % ± 0.31 %	1.46 % ± 0.28 %	0.02 % ± 0.04 %
M+2	0.43 % ± 0.18 %	1.13 % ± 0.11 %	4.11 % ± 0.18 %	2.43 % ± 0.24 %	0.00 % ± 0.00 %	0.36 % ± 0.12 %	0.43 % ± 0.17 %	0.11 % ± 0.09 %
M+3	5.23 % ± 0.21 %	0.15 % ± 0.04 %	0.30 % ± 0.10 %		0.00 % ± 0.00 %	12.10 % ± 0.24 %	0.00 % ± 0.00 %	0.01 % ± 0.02 %
M+4		0.00 % ± 0.00 %	0.12 % ± 0.03 %		0.13 % ± 0.03 %		0.00 % ± 0.00 %	0.00 % ± 0.00 %
M+5			0.03 % ± 0.01 %		0.00 % ± 0.00 %			0.09 % ± 0.04 %
M+6					0.00 % ± 0.00 %			0.00 % ± 0.01 %
M+7					0.00 % ± 0.00 %			0.00 % ± 0.00 %
M+8					0.00 % ± 0.00 %			0.00 % ± 0.00 %
M+9					0.00 % ± 0.00 %			0.08 % ± 0.11 %

3 mM [U-$^{13}C_3$]serine *Fno*								
	Alanine	Aspartate	Glutamate	Glycine	Phenylalanine	Serine	Threonine	Tyrosine
M+1	0.45 % ± 0.33 %	0.57 % ± 0.37 %	0.74 % ± 0.37 %	1.35 % ± 0.18 %	0.01 % ± 0.01 %	5.83 % ± 0.64 %	1.19 % ± 0.25 %	0.00 % ± 0.01 %
M+2	0.23 % ± 0.09 %	0.00 % ± 0.00 %	0.55 % ± 0.05 %	7.61 % ± 0.66 %	0.02 % ± 0.04 %	3.42 % ± 0.27 %	0.43 % ± 0.29 %	0.24 % ± 0.13 %
M+3	1.14 % ± 0.04 %	0.00 % ± 0.00 %	0.00 % ± 0.00 %		0.00 % ± 0.00 %	11.80 % ± 0.31 %	0.00 % ± 0.00 %	0.00 % ± 0.00 %
M+4		0.00 % ± 0.00 %	0.00 % ± 0.00 %		0.02 % ± 0.01 %		0.00 % ± 0.00 %	0.00 % ± 0.00 %
M+5			0.00 % ± 0.00 %		0.00 % ± 0.00 %			0.08 % ± 0.03 %
M+6					0.00 % ± 0.00 %			0.00 % ± 0.00 %
M+7					0.00 % ± 0.00 %			0.00 % ± 0.00 %
M+8					0.00 % ± 0.00 %			0.00 % ± 0.00 %
M+9					0.12 % ± 0.04 %			0.27 % ± 0.32 %

Supplemental Table S16 Relative fractions of isotopologues (mol%) of amino acids from experiments with *Fth* WT, *Fth* LVS and *Fno* grown in medium T supplemented with 25 mM [U-$^{13}C_3$]glycerol. M+x represents the mass of the unlabelled metabolite plus x labelled ^{13}C-atoms. Mean and SD from three independent experiments are shown.

25 mM [U-$^{13}C_3$]glycerol *Fth* WT								
	Alanine	Aspartate	Glutamate	Glycine	Phenylalanine	Serine	Threonine	Tyrosine
M+1	0.74 % ± 0.11 %	2.66 % ± 0.43 %	3.66 % ± 0.56 %	0.43 % ± 0.17 %	0.24 % ± 0.28 %	0.18 % ± 0.20 %	1.21 % ± 0.29 %	0.29 % ± 0.42 %
M+2	0.34 % ± 0.06 %	0.82 % ± 0.20 %	3.54 % ± 0.81 %	0.03 % ± 0.04 %	0.09 % ± 0.13 %	0.00 % ± 0.00 %	0.31 % ± 0.24 %	0.73 % ± 0.14 %
M+3	4.32 % ± 1.03 %	0.12 % ± 0.09 %	0.29 % ± 0.15 %		0.15 % ± 0.14 %	0.11 % ± 0.05 %	0.00 % ± 0.00 %	0.75 % ± 0.05 %
M+4		0.00 % ± 0.00 %	0.06 % ± 0.07 %		0.14 % ± 0.01 %		0.00 % ± 0.00 %	0.00 % ± 0.00 %
M+5			0.02 % ± 0.01 %		0.02 % ± 0.02 %			0.19 % ± 0.03 %
M+6					0.00 % ± 0.00 %			0.00 % ± 0.01 %
M+7					0.00 % ± 0.00 %			0.00 % ± 0.00 %
M+8					0.00 % ± 0.00 %			0.00 % ± 0.00 %
M+9					0.00 % ± 0.00 %			0.00 % ± 0.00 %

25 mM [U-$^{13}C_3$]glycerol *Fth* LVS								
	Alanine	Aspartate	Glutamate	Glycine	Phenylalanine	Serine	Threonine	Tyrosine
M+1	0.51 % ± 0.34 %	1.52 % ± 0.96 %	2.52 % ± 0.95 %	0.22 % ± 0.13 %	0.17 % ± 0.20 %	0.01 % ± 0.03 %	0.79 % ± 0.50 %	0.06 % ± 0.14 %
M+2	0.16 % ± 0.11 %	0.36 % ± 0.19 %	3.13 % ± 0.17 %	0.03 % ± 0.04 %	0.02 % ± 0.03 %	0.00 % ± 0.00 %	0.06 % ± 0.07 %	0.74 % ± 0.31 %
M+3	3.23 % ± 0.16 %	0.11 % ± 0.11 %	0.08 % ± 0.05 %		0.55 % ± 0.07 %	0.15 % ± 0.05 %	0.00 % ± 0.00 %	0.51 % ± 0.21 %
M+4		0.00 % ± 0.00 %	0.01 % ± 0.01 %		0.17 % ± 0.05 %		0.00 % ± 0.00 %	0.00 % ± 0.00 %
M+5			0.02 % ± 0.01 %		0.05 % ± 0.10 %			0.13 % ± 0.02 %
M+6					0.02 % ± 0.04 %			0.00 % ± 0.00 %
M+7					0.00 % ± 0.01 %			0.00 % ± 0.00 %
M+8					0.00 % ± 0.01 %			0.00 % ± 0.00 %
M+9					0.00 % ± 0.00 %			0.00 % ± 0.00 %

25 mM [U-$^{13}C_3$]glycerol *Fno*								
	Alanine	Aspartate	Glutamate	Glycine	Phenylalanine	Serine	Threonine	Tyrosine
M+1	1.24 % ± 0.35 %	1.49 % ± 0.96 %	6.71 % ± 0.91 %	1.05 % ± 0.21 %	0.13 % ± 0.19 %	1.68 % ± 0.66 %	1.15 % ± 0.71 %	0.37 % ± 0.53 %
M+2	1.29 % ± 0.22 %	0.97 % ± 0.53 %	13.09 % ± 1.62 %	1.77 % ± 0.46 %	0.03 % ± 0.08 %	0.79 % ± 0.30 %	0.41 % ± 0.51 %	4.95 % ± 0.29 %
M+3	21.27 % ± 2.36 %	0.40 % ± 0.19 %	2.50 % ± 0.76 %		0.00 % ± 0.00 %	1.47 % ± 0.61 %	0.00 % ± 0.00 %	5.64 % ± 0.62 %
M+4		0.03 % ± 0.04 %	1.22 % ± 0.47 %		0.11 % ± 0.04 %		0.00 % ± 0.00 %	0.14 % ± 0.11 %
M+5			0.20 % ± 0.09 %		0.03 % ± 0.05 %			2.96 % ± 0.36 %
M+6					0.01 % ± 0.03 %			0.42 % ± 0.16 %
M+7					0.00 % ± 0.01 %			0.02 % ± 0.05 %
M+8					0.11 % ± 0.12 %			0.10 % ± 0.07 %
M+9					0.01 % ± 0.01 %			0.01 % ± 0.02 %

Supplemental Table S17 Relative fractions of isotopologues (mol%) of polar metabolites from experiments with *Fth* WT, *Fth* LVS and *Fno* grown in medium T supplemented with 11 mM [U-$^{13}C_6$]glucose. M+x represents the mass of the unlabelled metabolite plus x labelled ^{13}C-atoms. Mean and SD from three independent experiments are shown.

11 mM [U-$^{13}C_6$]glucose *Fth* WT								
	Glycolic acid	Oxalate	Succinate	Fumarate	Malate	Aspartate	Glutamate	Citrate
M+1	0.65 % ± 0.19 %	0.22 % ± 0.15 %	4.88 % ± 0.66 %	0.59 % ± 0.91 %	11.30 % ± 2.55 %	0.75 % ± 0.21 %	8.71 % ± 0.38 %	0.03 % ± 0.08 %
M+2	10.06 % ± 0.75 %	2.44 % ± 0.26 %	3.68 % ± 0.36 %	0.71 % ± 0.96 %	4.24 % ± 1.17 %	0.06 % ± 0.09 %	8.81 % ± 0.41 %	0.17 % ± 0.46 %
M+3			0.89 % ± 0.08 %	0.53 % ± 0.19 %	1.06 % ± 1.03 %	0.25 % ± 0.05 %	1.03 % ± 0.12 %	0.04 % ± 0.09 %
M+4			0.00 % ± 0.01 %	0.18 % ± 0.20 %	0.26 % ± 0.51 %	0.01 % ± 0.02 %	0.33 % ± 0.03 %	0.36 % ± 0.61 %
M+5							0.06 % ± 0.01 %	0.41 % ± 0.80 %
M+6								0.11 % ± 0.22 %

11 mM [U-$^{13}C_6$]glucose *Fth* WT							
	Lactic acid	Alanine	Glycine	Glycerol	Serine	Threonine	Phenylalanine
M+1	0.00 % ± 0.00 %	2.82 % ± 0.18 %	0.43 % ± 0.05 %	1.82 % ± 0.33 %	0.47 % ± 0.28 %	0.01 % ± 0.03 %	0.44 % ± 0.27 %
M+2	0.55 % ± 0.09 %	1.96 % ± 0.04 %	0.05 % ± 0.06 %	1.08 % ± 0.10 %	0.48 % ± 0.17 %	0.29 % ± 0.09 %	0.71 % ± 0.18 %
M+3	6.95 % ± 0.84 %	7.71 % ± 0.53 %		6.83 % ± 0.43 %	0.06 % ± 0.06 %	0.03 % ± 0.06 %	1.12 % ± 0.15 %
M+4						0.03 % ± 0.04 %	0.76 % ± 0.08 %
M+5							0.20 % ± 0.02 %
M+6							0.10 % ± 0.01 %
M+7							0.06 % ± 0.01 %
M+8							0.00 % ± 0.00 %
M+9							0.00 % ± 0.00 %

11 mM [U-$^{13}C_6$]glucose *Fth* WT				
	3-Hydroxybutyric acid	Palmitic acid	Oleic acid	Stearic acid
M+1	2.63 % ± 0.73 %	2.55 % ± 0.27 %	5.34 % ± 0.33 %	3.80 % ± 0.18 %
M+2	15.58 % ± 1.28 %	22.35 % ± 0.70 %	24.63 % ± 1.17 %	22.79 % ± 0.99 %
M+3	0.43 % ± 0.62 %	1.78 % ± 0.06 %	2.78 % ± 0.16 %	2.87 % ± 0.18 %
M+4	0.99 % ± 0.52 %	7.67 % ± 0.38 %	7.75 % ± 0.67 %	10.22 % ± 0.98 %
M+5		0.55 % ± 0.09 %	1.01 % ± 0.31 %	1.19 % ± 0.25 %
M+6		1.94 % ± 0.48 %	2.20 % ± 0.88 %	3.45 % ± 1.27 %
M+7		0.12 % ± 0.08 %	0.38 % ± 0.19 %	0.41 % ± 0.26 %
M+8		0.41 % ± 0.30 %	0.63 % ± 0.54 %	1.09 % ± 0.90 %
M+9		0.03 % ± 0.04 %	0.09 % ± 0.09 %	0.14 % ± 0.15 %
M+10		0.07 % ± 0.11 %	0.20 % ± 0.18 %	0.36 % ± 0.41 %
M+11		0.00 % ± 0.01 %	0.03 % ± 0.03 %	0.04 % ± 0.05 %
M+12		0.02 % ± 0.03 %	0.04 % ± 0.05 %	0.07 % ± 0.12 %
M+13		0.04 % ± 0.02 %	0.01 % ± 0.01 %	0.01 % ± 0.01 %

M+14		0.01 % ± 0.01 %	0.02 % ± 0.02 %	0.01 % ± 0.02 %
M+15		0.01 % ± 0.01 %	0.01 % ± 0.01 %	0.01 % ± 0.01 %
M+16		0.00 % ± 0.01 %	0.02 % ± 0.02 %	0.00 % ± 0.00 %
M+17			0.01 % ± 0.01 %	0.04 % ± 0.03 %
M+18			0.01 % ± 0.01 %	0.01 % ± 0.01 %

11 mM [U-$^{13}C_6$]glucose *Fth* LVS								
	Glycolic acid	Oxalate	Succinate	Fumarate	Malate	Aspartate	Glutamate	Citrate
M+1	0.91 % ± 0.32 %	0.32 % ± 0.21 %	9.03 % ± 2.33 %	0.73 % ± 0.71 %	0.31 % ± 0.74 %	0.60 % ± 0.53 %	6.23 % ± 1.36 %	1.10 % ± 1.41 %
M+2	8.86 % ± 1.31 %	1.92 % ± 0.30 %	5.21 % ± 0.71 %	1.05 % ± 0.95 %	0.73 % ± 1.34 %	0.17 % ± 0.18 %	6.46 % ± 0.74 %	0.38 % ± 0.43 %
M+3			1.05 % ± 0.12 %	0.63 % ± 0.21 %	0.04 % ± 0.16 %	0.15 % ± 0.12 %	0.88 % ± 0.20 %	0.02 % ± 0.04 %
M+4			0.01 % ± 0.02 %	0.44 % ± 0.37 %	0.30 % ± 0.60 %	0.01 % ± 0.02 %	0.27 % ± 0.08 %	0.34 % ± 0.52 %
M+5							0.03 % ± 0.02 %	0.12 % ± 0.41 %
M+6								0.37 % ± 0.51 %

11 mM [U-$^{13}C_6$]glucose *Fth* LVS							
	Lactic acid	Alanine	Glycine	Glycerol	Serine	Threonine	Phenylalanine
M+1	0.07 % ± 0.11 %	2.56 % ± 0.50 %	0.15 % ± 0.18 %	1.38 % ± 0.36 %	0.31 % ± 0.26 %	0.00 % ± 0.02 %	0.54 % ± 0.37 %
M+2	0.88 % ± 0.19 %	1.90 % ± 0.27 %	0.12 % ± 0.19 %	0.85 % ± 0.18 %	0.47 % ± 0.31 %	0.24 % ± 0.29 %	1.76 % ± 0.34 %
M+3	7.06 % ± 1.36 %	7.77 % ± 0.47 %		6.61 % ± 0.83 %	0.04 % ± 0.06 %	0.03 % ± 0.06 %	2.14 % ± 0.36 %
M+4						0.02 % ± 0.04 %	1.44 % ± 0.16 %
M+5							0.35 % ± 0.05 %
M+6							0.25 % ± 0.03 %
M+7							0.14 % ± 0.02 %
M+8							0.00 % ± 0.00 %
M+9							0.00 % ± 0.00 %

11 mM [U-$^{13}C_6$]glucose *Fth* LVS				
	3-Hydroxybutyric acid	Palmitic acid	Oleic acid	Stearic acid
M+1	2.38 % ± 0.52 %	1.95 % ± 1.23 %	4.87 % ± 0.67 %	3.55 % ± 0.79 %
M+2	16.83 % ± 1.01 %	20.46 % ± 4.96 %	26.35 % ± 1.13 %	23.29 % ± 3.87 %
M+3	0.21 % ± 0.17 %	1.54 % ± 0.53 %	2.78 % ± 0.47 %	2.66 % ± 0.75 %
M+4	0.99 % ± 0.21 %	6.60 % ± 1.75 %	8.58 % ± 0.49 %	9.99 % ± 1.90 %
M+5		0.40 % ± 0.16 %	0.85 % ± 0.14 %	1.01 % ± 0.34 %
M+6		1.38 % ± 0.44 %	2.01 % ± 0.14 %	2.80 % ± 0.64 %
M+7		0.07 % ± 0.03 %	0.18 % ± 0.07 %	0.25 % ± 0.13 %
M+8		0.18 % ± 0.11 %	0.36 % ± 0.10 %	0.61 % ± 0.30 %
M+9		0.01 % ± 0.01 %	0.03 % ± 0.02 %	0.05 % ± 0.06 %
M+10		0.01 % ± 0.03 %	0.05 % ± 0.05 %	0.13 % ± 0.14 %
M+11		0.00 % ± 0.00 %	0.00 % ± 0.01 %	0.01 % ± 0.02 %
M+12		0.00 % ± 0.00 %	0.01 % ± 0.02 %	0.02 % ± 0.04 %
M+13		0.04 % ± 0.05 %	0.00 % ± 0.01 %	0.00 % ± 0.01 %
M+14		0.00 % ± 0.01 %	0.00 % ± 0.01 %	0.02 % ± 0.01 %

M+15		0.00 % ± 0.01 %	0.00 % ± 0.01 %	0.01 % ± 0.01 %
M+16		0.00 % ± 0.00 %	0.01 % ± 0.01 %	0.00 % ± 0.01 %
M+17			0.00 % ± 0.00 %	0.03 % ± 0.03 %
M+18			0.00 % ± 0.00 %	0.01 % ± 0.01 %

11 mM [U-$^{13}C_6$]glucose *Fno*								
	Glycolic acid	Oxalate	Succinate	Fumarate	Malate	Aspartate	Glutamate	Citrate
M+1	0.57 % ± 0.40 %	0.15 % ± 0.24 %	8.60 % ± 2.44 %	0.84 % ± 0.88 %	5.25 % ± 6.66 %	0.21 % ± 0.26 %	4.16 % ± 0.91 %	0.35 % ± 0.69 %
M+2	9.07 % ± 1.81 %	1.49 % ± 0.47 %	6.22 % ± 0.97 %	1.26 % ± 1.04 %	2.91 % ± 3.83 %	0.02 % ± 0.06 %	5.33 % ± 0.47 %	0.68 % ± 1.12 %
M+3			1.29 % ± 0.32 %	0.62 % ± 0.31 %	1.98 % ± 2.53 %	0.03 % ± 0.07 %	0.58 % ± 0.10 %	0.30 % ± 0.41 %
M+4			0.01 % ± 0.01 %	0.64 % ± 0.31 %	0.38 % ± 0.91 %	0.00 % ± 0.00 %	0.17 % ± 0.06 %	0.08 % ± 0.14 %
M+5							0.03 % ± 0.01 %	0.04 % ± 0.08 %
M+6								0.13 % ± 0.38 %

11 mM [U-$^{13}C_6$]glucose *Fno*							
	Lactic acid	Alanine	Glycine	Glycerol	Serine	Threonine	Phenylalanine
M+1	0.18 % ± 0.24 %	1.12 % ± 0.33 %	0.25 % ± 0.21 %	1.15 % ± 0.44 %	0.53 % ± 0.40 %	0.08 % ± 0.17 %	0.08 % ± 0.24 %
M+2	0.86 % ± 0.16 %	1.15 % ± 0.17 %	0.55 % ± 0.25 %	1.01 % ± 0.40 %	0.51 % ± 0.31 %	0.29 % ± 0.19 %	0.00 % ± 0.01 %
M+3	7.17 % ± 0.59 %	8.86 % ± 0.77 %		8.23 % ± 1.23 %	0.04 % ± 0.08 %	0.01 % ± 0.03 %	0.02 % ± 0.03 %
M+4						0.01 % ± 0.02 %	0.22 % ± 0.01 %
M+5							0.06 % ± 0.02 %
M+6							0.00 % ± 0.00 %
M+7							0.01 % ± 0.01 %
M+8							0.05 % ± 0.06 %
M+9							0.00 % ± 0.00 %

11 mM [U-$^{13}C_6$]glucose *Fno*				
	3-Hydroxybutyric acid	Palmitic acid	Oleic acid	Stearic acid
M+1	1.82 % ± 0.47 %	1.41 % ± 0.62 %	4.30 % ± 0.24 %	2.84 % ± 0.29 %
M+2	19.85 % ± 1.00 %	23.29 % ± 3.84 %	28.33 % ± 0.96 %	26.31 % ± 2.63 %
M+3	0.27 % ± 0.13 %	1.23 % ± 0.21 %	2.64 % ± 0.26 %	2.32 % ± 0.23 %
M+4	1.30 % ± 0.20 %	8.27 % ± 1.37 %	10.21 % ± 0.90 %	12.24 % ± 1.20 %
M+5		0.32 % ± 0.07 %	0.84 % ± 0.09 %	0.92 % ± 0.12 %
M+6		1.82 % ± 0.36 %	2.52 % ± 0.37 %	3.44 % ± 0.49 %
M+7		0.05 % ± 0.02 %	0.17 % ± 0.05 %	0.22 % ± 0.06 %
M+8		0.24 % ± 0.08 %	0.45 % ± 0.10 %	0.67 % ± 0.16 %
M+9		0.01 % ± 0.01 %	0.03 % ± 0.01 %	0.03 % ± 0.02 %
M+10		0.01 % ± 0.01 %	0.06 % ± 0.03 %	0.10 % ± 0.06 %
M+11		0.00 % ± 0.00 %	0.00 % ± 0.00 %	0.00 % ± 0.00 %
M+12		0.00 % ± 0.00 %	0.01 % ± 0.01 %	0.00 % ± 0.00 %
M+13		0.02 % ± 0.01 %	0.00 % ± 0.00 %	0.00 % ± 0.00 %

M+14		0.00 % ± 0.00 %	0.01 % ± 0.01 %	0.00 % ± 0.01 %
M+15		0.01 % ± 0.01 %	0.00 % ± 0.00 %	0.00 % ± 0.00 %
M+16		0.00 % ± 0.00 %	0.00 % ± 0.01 %	0.00 % ± 0.00 %
M+17			0.00 % ± 0.01 %	0.00 % ± 0.01 %
M+18			0.00 % ± 0.01 %	0.00 % ± 0.00 %

Supplemental Table S18 Relative fractions of isotopologues (mol%) of polar metabolites from experiments with *Fth* WT grown in medium T supplemented with 11 mM [1,2-$^{13}C_2$]glucose. M+x represents the mass of the unlabelled metabolite plus x labelled ^{13}C-atoms. Mean and SD from two independent experiments are shown.

11 mM [1,2-$^{13}C_2$]glucose *Fth* WT								
	Glycolic acid	Oxalate	Succinate	Fumarate	Malate	Aspartate	Glutamate	Citrate
M+1	0.94 % ± 0.30 %	0.00 % ± 0.00 %	0.00 % ± 0.00 %	0.00 % ± 0.00 %	0.78 % ± 0.88 %	0.53 % ± 0.31 %	3.63 % ± 0.29 %	0.00 % ± 0.00 %
M+2	4.58 % ± 0.26 %	0.20 % ± 0.15 %	0.74 % ± 0.22 %	0.15 % ± 0.16 %	1.24 % ± 0.33 %	0.00 % ± 0.00 %	3.16 % ± 0.42 %	0.52 % ± 1.22 %
M+3			0.37 % ± 0.02 %	0.34 % ± 0.33 %	0.29 % ± 0.45 %	0.08 % ± 0.05 %	0.01 % ± 0.02 %	1.10 % ± 1.49 %
M+4			0.01 % ± 0.01 %	0.02 % ± 0.05 %	0.18 % ± 0.24 %	0.00 % ± 0.00 %	0.02 % ± 0.01 %	0.23 % ± 0.50 %
M+5							0.03 % ± 0.01 %	0.06 % ± 0.14 %
M+6								0.34 % ± 0.52 %

11 mM [1,2-$^{13}C_2$]glucose *Fth* WT							
	Lactic acid	Alanine	Glycine	Glycerol	Serine	Threonine	Phenylalanine
M+1	0.00 % ± 0.00 %	1.10 % ± 0.16 %	0.22 % ± 0.10 %	0.74 % ± 0.25 %	0.38 % ± 0.22 %	0.00 % ± 0.00 %	0.30 % ± 0.20 %
M+2	7.50 % ± 0.61 %	4.00 % ± 0.33 %	0.02 % ± 0.03 %	3.55 % ± 0.37 %	0.40 % ± 0.13 %	0.31 % ± 0.12 %	1.42 % ± 0.26 %
M+3	0.04 % ± 0.04 %	0.12 % ± 0.01 %		0.12 % ± 0.04 %	0.04 % ± 0.06 %	0.02 % ± 0.04 %	0.03 % ± 0.02 %
M+4						0.01 % ± 0.01 %	0.24 % ± 0.01 %
M+5							0.03 % ± 0.01 %
M+6							0.00 % ± 0.00 %
M+7							0.00 % ± 0.00 %
M+8							0.00 % ± 0.00 %
M+9							0.00 % ± 0.00 %

11 mM [1,2-$^{13}C_2$]glucose *Fth* WT				
	3-Hydroxybutyric acid	Palmitic acid	Oleic acid	Stearic acid
M+1	1.25 % ± 0.39 %	0.89 % ± 0.22 %	3.79 % ± 0.35 %	3.58 % ± 0.34 %
M+2	5.66 % ± 0.83 %	15.27 % ± 1.20 %	18.24 % ± 1.48 %	18.86 % ± 1.36 %
M+3	0.13 % ± 0.18 %	0.51 % ± 0.12 %	1.17 % ± 0.28 %	1.36 % ± 0.33 %
M+4	0.09 % ± 0.19 %	2.53 % ± 0.53 %	2.97 % ± 0.74 %	4.20 % ± 0.92 %
M+5		0.08 % ± 0.03 %	0.21 % ± 0.11 %	0.34 % ± 0.13 %
M+6		0.30 % ± 0.13 %	0.43 % ± 0.21 %	0.73 % ± 0.29 %
M+7		0.02 % ± 0.01 %	0.04 % ± 0.02 %	0.05 % ± 0.04 %
M+8		0.02 % ± 0.03 %	0.07 % ± 0.04 %	0.13 % ± 0.07 %
M+9		0.01 % ± 0.01 %	0.01 % ± 0.01 %	0.01 % ± 0.01 %
M+10		0.00 % ± 0.00 %	0.05 % ± 0.03 %	0.03 % ± 0.02 %
M+11		0.00 % ± 0.00 %	0.01 % ± 0.01 %	0.00 % ± 0.00 %
M+12		0.00 % ± 0.00 %	0.01 % ± 0.01 %	0.00 % ± 0.00 %
M+13		0.04 % ± 0.02 %	0.01 % ± 0.01 %	0.00 % ± 0.00 %

M+14		0.00 % ± 0.00 %	0.01 % ± 0.01 %	0.02 % ± 0.02 %
M+15		0.02 % ± 0.01 %	0.01 % ± 0.00 %	0.01 % ± 0.01 %
M+16		0.00 % ± 0.00 %	0.01 % ± 0.02 %	0.01 % ± 0.01 %
M+17			0.01 % ± 0.02 %	0.03 % ± 0.01 %
M+18			0.02 % ± 0.02 %	0.01 % ± 0.01 %

Supplemental Table S19 Relative fractions of isotopologues (mol%) of polar metabolites from experiments with *Fth* WT, *Fth* LVS and *Fno* grown in medium T supplemented with 3 mM [U-$^{13}C_3$]serine. M+x represents the mass of the unlabelled metabolite plus x labelled ^{13}C-atoms. Mean and SD from three independent experiments are shown.

3 mM [U-$^{13}C_3$]serine *Fth* WT								
	Glycolic acid	Oxalate	Succinate	Fumarate	Malate	Aspartate	Glutamate	Citrate
M+1	0.06 % ± 0.11 %	0.00 % ± 0.00 %	0.00 % ± 0.00 %	0.00 % ± 0.00 %	0.78 % ± 0.93 %	0.38 % ± 0.39 %	0.92 % ± 0.34 %	0.09 % ± 0.22 %
M+2	0.34 % ± 0.23 %	0.00 % ± 0.00 %	0.00 % ± 0.00 %	0.00 % ± 0.00 %	1.38 % ± 0.53 %	0.00 % ± 0.00 %	0.81 % ± 0.05 %	0.03 % ± 0.06 %
M+3			0.20 % ± 0.03 %	0.42 % ± 0.22 %	0.00 % ± 0.00 %	0.02 % ± 0.04 %	0.00 % ± 0.01 %	0.18 % ± 0.45 %
M+4			0.01 % ± 0.01 %	0.04 % ± 0.09 %	0.80 % ± 1.11 %	0.00 % ± 0.00 %	0.00 % ± 0.00 %	0.00 % ± 0.00 %
M+5							0.01 % ± 0.01 %	0.13 % ± 0.25 %
M+6								0.24 % ± 0.52 %

3 mM [U-$^{13}C_3$]serine *Fth* WT							
	Lactic acid	Alanine	Glycine	Glycerol	Serine	Threonine	Phenylalanine
M+1	0.00 % ± 0.00 %	0.39 % ± 0.20 %	0.46 % ± 0.20 %	0.62 % ± 0.71 %	0.03 % ± 0.08 %	0.38 % ± 0.50 %	0.42 % ± 0.34 %
M+2	0.01 % ± 0.02 %	0.13 % ± 0.05 %	1.44 % ± 0.03 %	0.03 % ± 0.04 %	0.62 % ± 0.21 %	0.01 % ± 0.02 %	0.00 % ± 0.00 %
M+3	0.11 % ± 0.05 %	0.63 % ± 0.04 %		0.04 % ± 0.01 %	9.15 % ± 0.96 %	0.02 % ± 0.03 %	0.00 % ± 0.00 %
M+4						0.02 % ± 0.03 %	0.11 % ± 0.03 %
M+5							0.07 % ± 0.05 %
M+6							0.02 % ± 0.03 %
M+7							0.03 % ± 0.04 %
M+8							0.00 % ± 0.00 %
M+9							0.00 % ± 0.00 %

3 mM [U-$^{13}C_3$]serine *Fth* WT				
	3-Hydroxybutyric acid	Palmitic acid	Oleic acid	Stearic acid
M+1	2.00 % ± 0.27 %	2.59 % ± 0.57 %	6.70 % ± 0.26 %	4.43 % ± 0.47 %
M+2	3.49 % ± 0.45 %	13.23 % ± 1.33 %	21.11 % ± 0.54 %	9.79 % ± 1.81 %
M+3	0.21 % ± 0.29 %	2.56 % ± 0.48 %	5.19 % ± 1.17 %	1.49 % ± 0.08 %
M+4	0.50 % ± 0.27 %	5.09 % ± 0.74 %	9.87 % ± 2.16 %	2.30 % ± 0.23 %
M+5		1.20 % ± 0.27 %	2.81 % ± 0.91 %	0.51 % ± 0.08 %
M+6		1.65 % ± 0.34 %	3.82 % ± 1.19 %	0.63 % ± 0.08 %
M+7		0.34 % ± 0.08 %	0.99 % ± 0.35 %	0.15 % ± 0.06 %
M+8		0.37 % ± 0.07 %	1.06 % ± 0.36 %	0.15 % ± 0.04 %
M+9		0.05 % ± 0.02 %	0.22 % ± 0.09 %	0.02 % ± 0.02 %
M+10		0.04 % ± 0.01 %	0.18 % ± 0.06 %	0.01 % ± 0.01 %
M+11		0.01 % ± 0.02 %	0.01 % ± 0.01 %	0.01 % ± 0.01 %
M+12		0.05 % ± 0.08 %	0.02 % ± 0.02 %	0.03 % ± 0.05 %
M+13		0.04 % ± 0.03 %	0.01 % ± 0.03 %	0.01 % ± 0.01 %

M+14		0.01 % ± 0.02 %	0.03 % ± 0.03 %	0.11 % ± 0.17 %
M+15		0.01 % ± 0.02 %	0.01 % ± 0.01 %	0.05 % ± 0.07 %
M+16		0.01 % ± 0.01 %	0.01 % ± 0.01 %	0.02 % ± 0.04 %
M+17			0.00 % ± 0.00 %	0.07 % ± 0.04 %
M+18			0.01 % ± 0.01 %	0.03 % ± 0.05 %

3 mM [U-$^{13}C_3$]serine *Fth* LVS								
	Glycolic acid	Oxalate	Succinate	Fumarate	Malate	Aspartate	Glutamate	Citrate
M+1	0.07 % ± 0.16 %	0.00 % ± 0.00 %	0.00 % ± 0.00 %	0.00 % ± 0.00 %	1.04 % ± 1.61 %	0.21 % ± 0.33 %	0.57 % ± 0.34 %	0.05 % ± 0.13 %
M+2	0.30 % ± 0.24 %	0.00 % ± 0.00 %	0.00 % ± 0.00 %	0.00 % ± 0.00 %	1.56 % ± 2.82 %	0.00 % ± 0.00 %	0.67 % ± 0.15 %	0.09 % ± 0.21 %
M+3			0.18 % ± 0.05 %	0.42 % ± 0.30 %	1.16 % ± 1.48 %	0.02 % ± 0.05 %	0.00 % ± 0.00 %	0.60 % ± 1.46 %
M+4			0.00 % ± 0.00 %	0.11 % ± 0.18 %	1.12 % ± 1.05 %	0.01 % ± 0.03 %	0.00 % ± 0.00 %	0.32 % ± 0.55 %
M+5							0.01 % ± 0.01 %	0.13 % ± 0.32 %
M+6								0.48 % ± 1.19 %

3 mM [U-$^{13}C_3$]serine *Fth* LVS							
	Lactic acid	Alanine	Glycine	Glycerol	Serine	Threonine	Phenylalanine
M+1	0.00 % ± 0.00 %	0.30 % ± 0.15 %	0.26 % ± 0.08 %	0.60 % ± 0.73 %	0.02 % ± 0.05 %	0.41 % ± 0.38 %	0.32 % ± 0.19 %
M+2	0.00 % ± 0.00 %	0.10 % ± 0.03 %	1.15 % ± 0.05 %	0.03 % ± 0.04 %	0.33 % ± 0.23 %	0.05 % ± 0.12 %	0.00 % ± 0.00 %
M+3	0.12 % ± 0.03 %	0.80 % ± 0.06 %		0.04 % ± 0.05 %	5.48 % ± 0.23 %	0.05 % ± 0.06 %	0.05 % ± 0.06 %
M+4						0.01 % ± 0.02 %	0.14 % ± 0.01 %
M+5							0.04 % ± 0.02 %
M+6							0.00 % ± 0.00 %
M+7							0.00 % ± 0.00 %
M+8							0.00 % ± 0.00 %
M+9							0.00 % ± 0.00 %

3 mM [U-$^{13}C_3$]serine *Fth* LVS				
	3-Hydroxybutyric acid	Palmitic acid	Oleic acid	Stearic acid
M+1	1.10 % ± 0.44 %	2.20 % ± 0.28 %	5.70 % ± 0.44 %	4.09 % ± 0.43 %
M+2	3.86 % ± 0.46 %	14.56 % ± 0.80 %	20.30 % ± 0.58 %	9.97 % ± 1.48 %
M+3	0.01 % ± 0.02 %	2.07 % ± 0.16 %	4.32 % ± 0.44 %	1.23 % ± 0.16 %
M+4	0.41 % ± 0.50 %	5.61 % ± 0.55 %	9.71 % ± 1.33 %	2.24 % ± 0.36 %
M+5		1.01 % ± 0.16 %	2.34 % ± 0.41 %	0.42 % ± 0.05 %
M+6		1.98 % ± 0.26 %	4.01 % ± 0.82 %	0.67 % ± 0.07 %
M+7		0.31 % ± 0.04 %	0.92 % ± 0.21 %	0.11 % ± 0.02 %
M+8		0.47 % ± 0.08 %	1.22 % ± 0.28 %	0.15 % ± 0.03 %
M+9		0.05 % ± 0.02 %	0.23 % ± 0.07 %	0.01 % ± 0.01 %
M+10		0.06 % ± 0.02 %	0.22 % ± 0.07 %	0.01 % ± 0.02 %
M+11		0.02 % ± 0.02 %	0.04 % ± 0.02 %	0.01 % ± 0.01 %
M+12		0.02 % ± 0.03 %	0.03 % ± 0.02 %	0.02 % ± 0.03 %
M+13		0.03 % ± 0.02 %	0.01 % ± 0.01 %	0.01 % ± 0.01 %
M+14		0.00 % ± 0.00 %	0.02 % ± 0.02 %	0.07 % ± 0.12 %

M+15		0.00 % ± 0.01 %	0.01 % ± 0.02 %	0.04 % ± 0.04 %
M+16		0.00 % ± 0.00 %	0.00 % ± 0.00 %	0.02 % ± 0.02 %
M+17			0.00 % ± 0.01 %	0.04 % ± 0.03 %
M+18			0.01 % ± 0.01 %	0.03 % ± 0.03 %

3 mM [U-$^{13}C_3$]serine *Fno*								
	Glycolic acid	Oxalate	Succinate	Fumarate	Malate	Aspartate	Glutamate	Citrate
M+1	0.00 % ± 0.00 %	0.00 % ± 0.00 %	0.00 % ± 0.00 %	0.00 % ± 0.00 %	0.97 % ± 2.36 %	0.33 % ± 0.30 %	0.32 % ± 0.25 %	0.00 % ± 0.00 %
M+2	0.09 % ± 0.08 %	0.00 % ± 0.00 %	0.00 % ± 0.00 %	0.00 % ± 0.00 %	2.64 % ± 4.52 %	0.00 % ± 0.00 %	0.50 % ± 0.08 %	0.10 % ± 0.25 %
M+3			0.26 % ± 0.01 %	0.88 % ± 0.85 %	0.36 % ± 0.81 %	0.03 % ± 0.05 %	0.01 % ± 0.02 %	0.12 % ± 0.30 %
M+4			0.01 % ± 0.01 %	0.06 % ± 0.11 %	3.99 % ± 6.61 %	0.03 % ± 0.06 %	0.00 % ± 0.00 %	0.42 % ± 0.79 %
M+5							0.00 % ± 0.00 %	0.17 % ± 0.30 %
M+6								0.00 % ± 0.00 %

3 mM [U-$^{13}C_3$]serine *Fno*							
	Lactic acid	Alanine	Glycine	Glycerol	Serine	Threonine	Phenylalanine
M+1	0.90 % ± 0.19 %	1.14 % ± 0.08 %	0.84 % ± 0.10 %	0.82 % ± 0.47 %	0.22 % ± 0.16 %	0.50 % ± 0.63 %	0.25 % ± 0.15 %
M+2	1.23 % ± 0.08 %	0.85 % ± 0.03 %	8.06 % ± 0.15 %	0.04 % ± 0.10 %	0.99 % ± 0.21 %	0.00 % ± 0.00 %	0.00 % ± 0.00 %
M+3	4.58 % ± 0.45 %	4.78 % ± 0.44 %		0.04 % ± 0.06 %	19.65 % ± 0.54 %	0.04 % ± 0.05 %	0.02 % ± 0.05 %
M+4						0.03 % ± 0.03 %	0.14 % ± 0.02 %
M+5							0.07 % ± 0.05 %
M+6							0.02 % ± 0.03 %
M+7							0.00 % ± 0.00 %
M+8							0.14 % ± 0.11 %
M+9							0.00 % ± 0.00 %

3 mM [U-$^{13}C_3$]serine *Fno*				
	3-Hydroxybutyric acid	Palmitic acid	Oleic acid	Stearic acid
M+1	0.99 % ± 0.17 %	0.14 % ± 0.25 %	3.83 % ± 0.77 %	2.87 % ± 0.82 %
M+2	1.18 % ± 0.13 %	4.20 % ± 1.58 %	11.92 % ± 3.52 %	5.55 % ± 3.02 %
M+3	0.00 % ± 0.00 %	0.30 % ± 0.16 %	1.65 % ± 0.58 %	0.57 % ± 0.35 %
M+4	0.00 % ± 0.00 %	0.48 % ± 0.32 %	3.69 % ± 1.42 %	0.98 % ± 0.70 %
M+5		0.06 % ± 0.05 %	0.82 % ± 0.38 %	0.17 % ± 0.11 %
M+6		0.10 % ± 0.07 %	1.36 % ± 0.48 %	0.25 % ± 0.14 %
M+7		0.02 % ± 0.03 %	0.31 % ± 0.12 %	0.03 % ± 0.03 %
M+8		0.00 % ± 0.00 %	0.41 % ± 0.14 %	0.05 % ± 0.03 %
M+9		0.01 % ± 0.02 %	0.06 % ± 0.03 %	0.01 % ± 0.02 %
M+10		0.00 % ± 0.00 %	0.04 % ± 0.04 %	0.01 % ± 0.01 %
M+11		0.00 % ± 0.01 %	0.00 % ± 0.00 %	0.01 % ± 0.01 %
M+12		0.01 % ± 0.02 %	0.01 % ± 0.01 %	0.00 % ± 0.00 %
M+13		0.06 % ± 0.01 %	0.01 % ± 0.01 %	0.00 % ± 0.00 %

M+14		0.01 % ± 0.02 %	0.02 % ± 0.02 %	0.00 % ± 0.00 %
M+15		0.03 % ± 0.02 %	0.02 % ± 0.02 %	0.00 % ± 0.00 %
M+16		0.01 % ± 0.01 %	0.03 % ± 0.03 %	0.00 % ± 0.00 %
M+17			0.01 % ± 0.01 %	0.01 % ± 0.01 %
M+18			0.01 % ± 0.02 %	0.01 % ± 0.02 %

Supplemental Table S20 Relative fractions of isotopologues (mol%) of polar metabolites from experiments with *Fth* WT, *Fth* LVS and *Fno* grown in medium T supplemented with 25 mM [U-$^{13}C_3$]glycerol. M+x represents the mass of the unlabelled metabolite plus x labelled ^{13}C-atoms. Mean and SD from three independent experiments are shown.

25 mM [U-$^{13}C_3$]glycerol *Fth* WT								
	Glycolic acid	Oxalate	Succinate	Fumarate	Malate	Aspartate	Glutamate	Citrate
M+1	1.37 % ± 1.44 %	1.71 % ± 1.89 %	4.23 % ± 3.95 %	12.38 % ± 13.57 %	8.87 % ± 6.87 %	1.68 % ± 1.66 %	4.92 % ± 2.40 %	1.30 % ± 2.02 %
M+2	5.42 % ± 3.84 %	0.28 % ± 0.31 %	9.19 % ± 8.99 %	2.31 % ± 1.95 %	2.38 % ± 1.29 %	0.03 % ± 0.06 %	4.56 % ± 1.57 %	0.58 % ± 1.39 %
M+3			1.10 % ± 0.80 %	0.34 % ± 0.36 %	0.31 % ± 0.64 %	0.14 % ± 0.10 %	0.27 % ± 0.27 %	0.14 % ± 0.31 %
M+4			0.28 % ± 0.27 %	0.11 % ± 0.21 %	0.30 % ± 0.38 %	0.01 % ± 0.02 %	0.12 % ± 0.04 %	0.19 % ± 0.45 %
M+5							0.03 % ± 0.02 %	0.37 % ± 0.58 %
M+6								0.21 % ± 0.35 %

25 mM [U-$^{13}C_3$]glycerol *Fth* WT							
	Lactic acid	Alanine	Glycine	Glycerol	Serine	Threonine	Phenylalanine
M+1	0.03 % ± 0.08 %	1.19 % ± 0.74 %	2.21 % ± 2.41 %	0.05 % ± 0.01 %	0.82 % ± 0.52 %	0.33 % ± 0.50 %	0.26 % ± 0.20 %
M+2	0.14 % ± 0.21 %	0.31 % ± 0.15 %	0.29 % ± 0.28 %	2.44 % ± 0.09 %	0.47 % ± 0.27 %	0.22 % ± 0.19 %	0.57 % ± 0.19 %
M+3	1.11 % ± 0.14 %	4.13 % ± 1.55 %		91.33 % ± 0.20 %	0.05 % ± 0.06 %	0.04 % ± 0.09 %	1.10 % ± 0.15 %
M+4						0.03 % ± 0.05 %	0.14 % ± 0.02 %
M+5							0.22 % ± 0.08 %
M+6							0.06 % ± 0.06 %
M+7							0.01 % ± 0.01 %
M+8							0.00 % ± 0.00 %
M+9							0.00 % ± 0.00 %

25 mM [U-$^{13}C_3$]glycerol *Fth* WT				
	3-Hydroxybutyric acid	Palmitic acid	Oleic acid	Stearic acid
M+1	1.75 % ± 0.93 %	0.67 % ± 0.73 %	3.67 % ± 1.30 %	2.37 % ± 1.21 %
M+2	7.97 % ± 4.36 %	10.32 % ± 9.83 %	17.73 % ± 3.14 %	11.86 % ± 11.49 %
M+3	0.87 % ± 0.70 %	0.48 % ± 0.40 %	1.26 % ± 0.43 %	0.84 % ± 0.85 %
M+4	0.68 % ± 0.65 %	2.56 % ± 2.55 %	3.43 % ± 1.27 %	3.59 % ± 3.64 %
M+5		0.09 % ± 0.09 %	0.27 % ± 0.14 %	0.26 % ± 0.26 %
M+6		0.42 % ± 0.46 %	0.58 % ± 0.33 %	0.76 % ± 0.79 %
M+7		0.01 % ± 0.02 %	0.04 % ± 0.03 %	0.04 % ± 0.04 %
M+8		0.05 % ± 0.05 %	0.08 % ± 0.07 %	0.12 % ± 0.13 %
M+9		0.00 % ± 0.00 %	0.01 % ± 0.01 %	0.01 % ± 0.01 %
M+10		0.00 % ± 0.00 %	0.02 % ± 0.02 %	0.02 % ± 0.02 %
M+11		0.00 % ± 0.00 %	0.00 % ± 0.00 %	0.00 % ± 0.00 %
M+12		0.00 % ± 0.00 %	0.01 % ± 0.01 %	0.00 % ± 0.00 %
M+13		0.00 % ± 0.00 %	0.00 % ± 0.00 %	0.00 % ± 0.00 %

M+14		0.00 % ± 0.00 %	0.00 % ± 0.01 %	0.00 % ± 0.00 %
M+15		0.01 % ± 0.01 %	0.01 % ± 0.01 %	0.00 % ± 0.00 %
M+16		0.00 % ± 0.00 %	0.01 % ± 0.03 %	0.00 % ± 0.00 %
M+17			0.00 % ± 0.01 %	0.01 % ± 0.01 %
M+18			0.02 % ± 0.03 %	0.02 % ± 0.03 %

25 mM [U-$^{13}C_3$]glycerol *Fth* LVS								
	Glycolic acid	Oxalate	Succinate	Fumarate	Malate	Aspartate	Glutamate	Citrate
M+1	0.19 % ± 0.19 %	0.00 % ± 0.00 %	2.52 % ± 0.93 %	0.00 % ± 0.00 %	2.55 % ± 4.34 %	0.62 % ± 0.58 %	2.37 % ± 0.36 %	1.22 % ± 1.93 %
M+2	2.20 % ± 0.43 %	0.05 % ± 0.12 %	2.04 % ± 0.43 %	0.43 % ± 0.59 %	0.02 % ± 0.05 %	0.11 % ± 0.28 %	3.28 % ± 0.55 %	0.03 % ± 0.07 %
M+3			0.41 % ± 0.11 %	0.25 % ± 0.37 %	1.06 % ± 1.18 %	0.06 % ± 0.08 %	0.08 % ± 0.11 %	0.51 % ± 0.66 %
M+4			0.02 % ± 0.03 %	0.11 % ± 0.21 %	1.27 % ± 1.49 %	0.03 % ± 0.05 %	0.03 % ± 0.03 %	0.11 % ± 0.28 %
M+5							0.03 % ± 0.02 %	0.39 % ± 0.96 %
M+6								0.22 % ± 0.35 %

25 mM [U-$^{13}C_3$]glycerol *Fth* LVS							
	Lactic acid	Alanine	Glycine	Glycerol	Serine	Threonine	Phenylalanine
M+1	0.00 % ± 0.00 %	0.31 % ± 0.20 %	0.15 % ± 0.15 %	0.05 % ± 0.03 %	0.23 % ± 0.40 %	0.00 % ± 0.00 %	0.11 % ± 0.19 %
M+2	0.08 % ± 0.11 %	0.11 % ± 0.08 %	0.03 % ± 0.04 %	2.44 % ± 0.04 %	0.10 % ± 0.24 %	0.11 % ± 0.17 %	1.38 % ± 0.19 %
M+3	1.26 % ± 0.15 %	2.34 % ± 0.36 %		91.06 % ± 0.64 %	0.22 % ± 0.27 %	0.14 % ± 0.21 %	1.99 % ± 0.08 %
M+4						0.02 % ± 0.04 %	0.09 % ± 0.03 %
M+5							0.19 % ± 0.08 %
M+6							0.03 % ± 0.04 %
M+7							0.00 % ± 0.00 %
M+8							0.00 % ± 0.00 %
M+9							0.00 % ± 0.00 %

25 mM [U-$^{13}C_3$]glycerol *Fth* LVS				
	3-Hydroxybutyric acid	Palmitic acid	Oleic acid	Stearic acid
M+1	1.36 % ± 1.23 %	0.11 % ± 0.23 %	2.82 % ± 0.33 %	0.81 % ± 0.80 %
M+2	6.34 % ± 1.90 %	8.67 % ± 5.13 %	18.80 % ± 1.06 %	7.44 % ± 4.87 %
M+3	0.44 % ± 0.61 %	0.23 % ± 0.10 %	0.89 % ± 0.17 %	0.40 % ± 0.24 %
M+4	0.51 % ± 0.67 %	1.42 % ± 0.87 %	3.15 % ± 0.25 %	1.62 % ± 1.13 %
M+5		0.03 % ± 0.02 %	0.16 % ± 0.07 %	0.06 % ± 0.04 %
M+6		0.10 % ± 0.08 %	0.39 % ± 0.05 %	0.19 % ± 0.13 %
M+7		0.00 % ± 0.00 %	0.02 % ± 0.01 %	0.01 % ± 0.01 %
M+8		0.00 % ± 0.00 %	0.04 % ± 0.02 %	0.02 % ± 0.01 %
M+9		0.00 % ± 0.00 %	0.00 % ± 0.00 %	0.00 % ± 0.00 %
M+10		0.00 % ± 0.00 %	0.00 % ± 0.00 %	0.00 % ± 0.00 %
M+11		0.00 % ± 0.00 %	0.00 % ± 0.00 %	0.00 % ± 0.00 %
M+12		0.00 % ± 0.00 %	0.00 % ± 0.00 %	0.00 % ± 0.00 %
M+13		0.00 % ± 0.00 %	0.00 % ± 0.00 %	0.01 % ± 0.01 %
M+14		0.00 % ± 0.00 %	0.01 % ± 0.01 %	0.00 % ± 0.00 %

M+15		0.00 % ± 0.00 %	0.00 % ± 0.01 %	0.01 % ± 0.00 %
M+16		0.00 % ± 0.00 %	0.00 % ± 0.00 %	0.00 % ± 0.01 %
M+17			0.00 % ± 0.00 %	0.01 % ± 0.01 %
M+18			0.00 % ± 0.00 %	0.00 % ± 0.00 %

25 mM [U-$^{13}C_3$]glycerol *Fno*								
	Glycolic acid	Oxalate	Succinate	Fumarate	Malate	Aspartate	Glutamate	Citrate
M+1	1.50 % ± 1.97 %	1.12 % ± 1.61 %	12.89 % ± 2.33 %	9.50 % ± 10.42 %	10.01 % ± 9.01 %	1.24 % ± 1.45 %	5.64 % ± 1.64 %	2.23 % ± 1.81 %
M+2	5.00 % ± 2.37 %	0.28 % ± 0.42 %	12.23 % ± 3.10 %	4.43 % ± 3.17 %	7.84 % ± 6.44 %	0.04 % ± 0.13 %	9.21 % ± 1.65 %	0.58 % ± 0.94 %
M+3			4.80 % ± 1.87 %	2.10 % ± 1.41 %	1.60 % ± 2.29 %	0.10 % ± 0.14 %	2.45 % ± 0.83 %	2.71 % ± 6.45 %
M+4			0.73 % ± 0.66 %	0.87 % ± 0.90 %	5.38 % ± 7.05 %	0.06 % ± 0.11 %	1.16 % ± 0.49 %	1.05 % ± 2.19 %
M+5							0.25 % ± 0.15 %	0.39 % ± 0.75 %
M+6								2.64 % ± 5.83 %

25 mM [U-$^{13}C_3$]glycerol *Fno*							
	Lactic acid	Alanine	Glycine	Glycerol	Serine	Threonine	Phenylalanine
M+1	0.23 % ± 0.30 %	1.12 % ± 0.91 %	1.39 % ± 1.64 %	0.22 % ± 0.22 %	0.54 % ± 0.65 %	0.00 % ± 0.00 %	0.18 % ± 0.24 %
M+2	0.74 % ± 0.45 %	1.10 % ± 0.62 %	0.95 % ± 1.00 %	1.44 % ± 0.81 %	0.32 % ± 0.29 %	0.40 % ± 0.29 %	0.00 % ± 0.01 %
M+3	10.70 % ± 4.81 %	16.40 % ± 5.66 %		57.49 % ± 27.26 %	0.14 % ± 0.13 %	0.06 % ± 0.06 %	0.05 % ± 0.06 %
M+4						0.02 % ± 0.02 %	0.15 % ± 0.02 %
M+5							0.09 % ± 0.04 %
M+6							0.00 % ± 0.00 %
M+7							0.00 % ± 0.01 %
M+8							0.04 % ± 0.03 %
M+9							0.00 % ± 0.00 %

25 mM [U-$^{13}C_3$]glycerol *Fno*				
	3-Hydroxybutyric acid	Palmitic acid	Oleic acid	Stearic acid
M+1	2.80 % ± 0.49 %	1.16 % ± 0.88 %	3.28 % ± 0.33 %	1.28 % ± 0.55 %
M+2	38.28 % ± 2.61 %	15.24 % ± 5.09 %	19.76 % ± 0.75 %	11.15 % ± 4.40 %
M+3	1.16 % ± 0.52 %	2.96 % ± 1.36 %	4.87 % ± 1.25 %	2.04 % ± 0.99 %
M+4	7.70 % ± 1.84 %	14.61 % ± 6.33 %	16.51 % ± 4.03 %	11.45 % ± 5.94 %
M+5		2.43 % ± 1.23 %	4.17 % ± 1.32 %	2.21 % ± 1.27 %
M+6		9.16 % ± 4.57 %	10.70 % ± 3.41 %	9.13 % ± 5.59 %
M+7		1.20 % ± 0.65 %	2.28 % ± 0.84 %	1.54 % ± 1.03 %
M+8		3.88 % ± 2.26 %	4.93 % ± 1.94 %	5.15 % ± 3.52 %
M+9		0.38 % ± 0.24 %	0.87 % ± 0.37 %	0.75 % ± 0.56 %
M+10		1.10 % ± 0.77 %	1.61 % ± 0.74 %	2.07 % ± 1.60 %
M+11		0.03 % ± 0.04 %	0.21 % ± 0.11 %	0.25 % ± 0.22 %
M+12		0.17 % ± 0.15 %	0.38 % ± 0.21 %	0.57 % ± 0.49 %
M+13		0.01 % ± 0.01 %	0.04 % ± 0.03 %	0.05 % ± 0.05 %

M+14		0.01 % ± 0.02 %	0.07 % ± 0.05 %	0.08 % ± 0.08 %
M+15		0.01 % ± 0.02 %	0.00 % ± 0.01 %	0.00 % ± 0.00 %
M+16		0.00 % ± 0.00 %	0.01 % ± 0.02 %	0.00 % ± 0.00 %
M+17			0.00 % ± 0.01 %	0.00 % ± 0.00 %
M+18			0.02 % ± 0.02 %	0.00 % ± 0.00 %

Supplemental Table S21 Relative fractions of isotopologues (mol%) of sugars from experiments with *Fth* WT, *Fth* LVS and *Fno* grown in medium T supplemented with 11 mM [U-$^{13}C_6$]glucose. M+x represents the mass of the unlabelled metabolite plus x labelled ^{13}C-atoms. Mean and SD from three independent experiments are shown.

11 mM [U-$^{13}C_6$]glucose *Fth* WT				
	Glucose in glycogen	Free fructose	Muramic acid	Glucosamine
M+1	0.98 % ± 0.24 %	0.13 % ± 0.21 %	4.91 % ± 2.33 %	2.80 % ± 0.72 %
M+2	1.30 % ± 0.17 %	0.37 % ± 0.61 %	4.72 % ± 1.97 %	3.37 % ± 0.52 %
M+3	1.73 % ± 0.21 %	1.23 % ± 0.28 %	7.49 % ± 1.67 %	2.49 % ± 0.66 %
M+4	0.70 % ± 0.07 %	0.74 % ± 0.32 %	3.42 % ± 0.88 %	1.35 % ± 0.17 %
M+5	0.57 % ± 0.05 %	0.05 % ± 0.07 %	0.35 % ± 0.54 %	1.10 % ± 0.10 %
M+6	8.00 % ± 0.72 %	11.18 % ± 0.96 %	3.49 % ± 1.84 %	6.12 % ± 0.92 %

11 mM [U-$^{13}C_6$]glucose *Fth* LVS				
	Glucose in glycogen	Free fructose	Muramic acid	Glucosamine
M+1	2.58 % ± 2.36 %	0.04 % ± 0.14 %	6.21 % ± 2.44 %	3.87 % ± 2.34 %
M+2	1.74 % ± 0.19 %	0.39 % ± 0.43 %	4.58 % ± 2.74 %	3.02 % ± 0.50 %
M+3	1.98 % ± 0.31 %	1.09 % ± 0.70 %	7.45 % ± 2.61 %	1.97 % ± 0.60 %
M+4	1.01 % ± 0.06 %	0.38 % ± 0.43 %	3.47 % ± 2.07 %	1.36 % ± 0.17 %
M+5	0.54 % ± 0.04 %	0.04 % ± 0.08 %	0.54 % ± 1.03 %	1.05 % ± 0.29 %
M+6	7.48 % ± 0.62 %	10.74 % ± 0.63 %	2.87 % ± 1.61 %	5.75 % ± 0.78 %

11 mM [U-$^{13}C_6$]glucose *Fno*				
	Glucose in glycogen	Free fructose	Muramic acid	Glucosamine
M+1	0.16 % ± 0.23 %	0.30 % ± 0.65 %	5.47 % ± 3.92 %	3.17 % ± 2.26 %
M+2	0.61 % ± 0.24 %	1.02 % ± 0.83 %	2.87 % ± 2.45 %	2.81 % ± 0.75 %
M+3	0.61 % ± 0.19 %	1.70 % ± 0.71 %	4.66 % ± 3.53 %	1.69 % ± 0.70 %
M+4	0.41 % ± 0.12 %	0.93 % ± 0.47 %	3.18 % ± 1.87 %	1.47 % ± 0.35 %
M+5	0.52 % ± 0.12 %	0.22 % ± 0.36 %	0.14 % ± 0.44 %	0.98 % ± 0.31 %
M+6	8.44 % ± 0.88 %	10.13 % ± 0.54 %	3.62 % ± 1.44 %	6.69 % ± 0.97 %

Supplemental Table S22 Relative fractions of isotopologues (mol%) of sugars from experiments with *Fth* WT grown in medium T supplemented with 11 mM [1,2-$^{13}C_2$]glucose. M+x represents the mass of the unlabelled metabolite plus x labelled ^{13}C-atoms. Mean and SD from two independent experiments are shown.

11 mM [1,2-$^{13}C_2$]glucose *Fth* WT				
	Glucose in glycogen	Free fructose	Muramic acid	Glucosamine
M+1	0.24 % ± 0.24 %	0.00 % ± 0.00 %	2.19 % ± 1.94 %	1.46 % ± 0.88 %
M+2	13.30 % ± 0.52 %	11.31 % ± 0.78 %	18.83 % ± 1.09 %	14.15 % ± 0.55 %
M+3	0.01 % ± 0.01 %	0.66 % ± 0.73 %	0.00 % ± 0.01 %	0.02 % ± 0.07 %
M+4	0.08 % ± 0.03 %	1.19 % ± 0.28 %	1.55 % ± 0.33 %	0.01 % ± 0.02 %
M+5	0.00 % ± 0.01 %	0.00 % ± 0.00 %	0.12 % ± 0.18 %	0.33 % ± 0.08 %
M+6	0.01 % ± 0.01 %	0.00 % ± 0.00 %	0.07 % ± 0.11 %	0.00 % ± 0.00 %

Supplemental Table S23 Relative fractions of isotopologues (mol%) of sugars from experiments with *Fth* WT, *Fth* LVS and *Fno* grown in medium T supplemented with 3 mM [U-$^{13}C_3$]serine. M+x represents the mass of the unlabelled metabolite plus x labelled ^{13}C-atoms. Mean and SD from three independent experiments are shown.

3 mM [U-$^{13}C_3$]serine *Fth* WT				
	Glucose in glycogen	Free fructose	Muramic acid	Glucosamine
M+1	0.01 % ± 0.03 %	0.00 % ± 0.01 %	1.25 % ± 1.60 %	1.09 % ± 0.93 %
M+2	0.00 % ± 0.00 %	0.00 % ± 0.00 %	0.61 % ± 0.53 %	0.48 % ± 0.38 %
M+3	0.00 % ± 0.00 %	0.89 % ± 0.24 %	0.05 % ± 0.11 %	0.12 % ± 0.18 %
M+4	0.00 % ± 0.00 %	0.16 % ± 0.16 %	0.06 % ± 0.12 %	0.01 % ± 0.02 %
M+5	0.00 % ± 0.00 %	0.00 % ± 0.00 %	0.09 % ± 0.16 %	0.01 % ± 0.02 %
M+6	0.01 % ± 0.02 %	0.00 % ± 0.00 %	0.11 % ± 0.18 %	0.00 % ± 0.01 %

3 mM [U-$^{13}C_3$]serine *Fth* LVS				
	Glucose in glycogen	Free fructose	Muramic acid	Glucosamine
M+1	0.00 % ± 0.00 %	0.00 % ± 0.00 %	0.84 % ± 0.88 %	0.82 % ± 0.90 %
M+2	0.00 % ± 0.00 %	0.23 % ± 0.44 %	0.72 % ± 0.50 %	0.41 % ± 0.44 %
M+3	0.00 % ± 0.00 %	0.74 % ± 0.49 %	0.06 % ± 0.14 %	0.04 % ± 0.09 %
M+4	0.01 % ± 0.00 %	0.12 % ± 0.20 %	0.04 % ± 0.10 %	0.01 % ± 0.01 %
M+5	0.00 % ± 0.00 %	0.00 % ± 0.00 %	0.07 % ± 0.14 %	0.01 % ± 0.02 %
M+6	0.00 % ± 0.00 %	0.00 % ± 0.00 %	0.09 % ± 0.29 %	0.01 % ± 0.02 %

3 mM [U-$^{13}C_3$]serine *Fno*				
	Glucose in glycogen	Free fructose	Muramic acid	Glucosamine
M+1	0.00 % ± 0.00 %	0.07 % ± 0.17 %	3.66 % ± 3.30 %	1.23 % ± 0.79 %
M+2	0.00 % ± 0.00 %	0.08 % ± 0.20 %	0.39 % ± 0.84 %	0.50 % ± 0.42 %
M+3	0.00 % ± 0.00 %	0.56 % ± 0.45 %	0.37 % ± 0.76 %	0.01 % ± 0.04 %
M+4	0.00 % ± 0.00 %	0.21 % ± 0.19 %	0.35 % ± 0.58 %	0.00 % ± 0.00 %
M+5	0.00 % ± 0.00 %	0.00 % ± 0.00 %	0.20 % ± 0.53 %	0.01 % ± 0.03 %
M+6	0.00 % ± 0.00 %	0.00 % ± 0.00 %	0.51 % ± 0.84 %	0.00 % ± 0.01 %

Supplemental Table S24 Relative fractions of isotopologues (mol%) of sugars from experiments with *Fth* WT, *Fth* LVS and *Fno* grown in medium T supplemented with 25 mM [U-$^{13}C_3$]glycerol. M+x represents the mass of the unlabelled metabolite plus x labelled ^{13}C-atoms. Mean and SD from three independent experiments are shown.

25 mM [U-$^{13}C_3$]glycerol *Fth* WT				
	Glucose in glycogen	Free fructose	Muramic acid	Glucosamine
M+1	0.00 % ± 0.00 %	0.05 % ± 0.08 %	2.46 % ± 2.50 %	0.89 % ± 0.67 %
M+2	0.02 % ± 0.04 %	0.00 % ± 0.00 %	0.81 % ± 1.14 %	0.38 % ± 0.56 %
M+3	1.12 % ± 0.12 %	0.89 % ± 0.65 %	6.26 % ± 2.14 %	1.76 % ± 0.43 %
M+4	0.01 % ± 0.02 %	0.52 % ± 0.44 %	1.20 % ± 1.05 %	0.03 % ± 0.05 %
M+5	0.01 % ± 0.01 %	0.00 % ± 0.00 %	0.13 % ± 0.35 %	0.11 % ± 0.07 %
M+6	0.00 % ± 0.00 %	0.00 % ± 0.00 %	0.26 % ± 0.38 %	0.07 % ± 0.07 %

25 mM [U-$^{13}C_3$]glycerol *Fth* LVS				
	Glucose in glycogen	Free fructose	Muramic acid	Glucosamine
M+1	0.04 % ± 0.07 %	0.00 % ± 0.00 %	1.06 % ± 1.43 %	0.63 % ± 0.63 %
M+2	0.00 % ± 0.00 %	0.01 % ± 0.03 %	0.93 % ± 0.72 %	0.50 % ± 0.48 %
M+3	0.79 % ± 0.07 %	1.09 % ± 0.47 %	4.37 % ± 1.17 %	1.19 % ± 0.24 %
M+4	0.01 % ± 0.01 %	0.06 % ± 0.10 %	0.77 % ± 0.67 %	0.02 % ± 0.04 %
M+5	0.01 % ± 0.01 %	0.00 % ± 0.00 %	0.02 % ± 0.07 %	0.02 % ± 0.03 %
M+6	0.00 % ± 0.00 %	0.00 % ± 0.00 %	0.29 % ± 0.64 %	0.06 % ± 0.13 %

25 mM [U-$^{13}C_3$]glycerol *Fno*				
	Glucose in glycogen	Free fructose	Muramic acid	Glucosamine
M+1	0.01 % ± 0.03 %	0.48 % ± 0.69 %	3.63 % ± 5.55 %	1.81 % ± 2.11 %
M+2	0.12 % ± 0.08 %	0.27 % ± 0.52 %	2.75 % ± 4.68 %	0.70 % ± 0.55 %
M+3	1.66 % ± 0.31 %	1.96 % ± 0.56 %	21.72 % ± 11.85 %	4.52 % ± 1.27 %
M+4	0.04 % ± 0.02 %	0.46 % ± 0.43 %	2.27 % ± 1.62 %	0.33 % ± 0.90 %
M+5	0.03 % ± 0.01 %	0.00 % ± 0.00 %	2.93 % ± 5.45 %	0.29 % ± 0.42 %
M+6	0.15 % ± 0.04 %	0.00 % ± 0.00 %	0.75 % ± 1.19 %	0.20 % ± 0.24 %

Supplemental Table S25 Composition of amino acids, polar metabolites and glucose in medium T. Mean and SD from two independent experiments are shown.

Amino acids in Medium T umol/mL	
Ala	5.01 ± 0.38
Gly	5.75 ± 0.33
Val	5.36 ± 0.80
Nor	1.00 ± 0.00
Leu	6.43 ± 1.28
Ile	3.45 ± 0.69
Pro	8.96 ± 1.30
Met	0.29 ± 0.20
Ser	3.06 ± 0.66
Thr	0.76 ± 0.19
Phe	1.90 ± 0.57
Asp	7.54 ± 1.32
Glu	14.64 ± 2.79
Lys	4.07 ± 1.15
His	0.49 ± 0.21
Tyr	0.91 ± 0.18

Polar metabolites in Medium T umol/mL	
Lactate	0.07 ± 0.00
Glycolic acid	0.01 ± 0.00
Ala	0.47 ± 0.01
Gly	0.19 ± 0.00
Val	0.47 ± 0.01
Leu	1.11 ± 0.02
Ile	0.31 ± 0.01
Pro	0.53 ± 0.02
5-oxo-Pro	0.74 ± 0.02
Met	0.18 ± 0.01
Ser	0.53 ± 0.04
Thr	0.25 ± 0.03
Phe	0.40 ± 0.01
Asp	0.70 ± 0.01
Glu	1.04 ± 0.02
Asn	0.26 ± 0.00
Lys	0.49 ± 0.01
Citrate	0.89 ± 0.02
Tyr	0.13 ± 0.00

Glucose in Medium T umol/mL	
Glucose	75.31 ± 9.42

Supplemental Table S26 Composition of medium T in the [U-$^{13}C_6$]glucose, [1,2-$^{13}C_2$]glucose, [U-$^{13}C_3$]serine, or [U-$^{13}C_3$]glycerol labelled experiments.

[U-$^{13}C_6$] Glucose Medium T	
brain heart infusion broth (Fa. Difco)	10 g/L
bacto trypton (Fa. Difco)	10 g/L
technical casamino acid (Fa. Difco)	10 g/L
$MgSO_4$	0.05 g/L
$FeSO_4$	0.1 g/L
NaCitrat	1.2 g/L
KCl	0.2 g/L
K_2HPO_4	0.4 g/L
L-Cystein	0.6 g/L
Glucose	15 g/L
U-$^{13}C_6$-Glucose	2 g/L

[1,2-$^{13}C_2$] Glucose Medium T	
brain heart infusion broth (Fa. Difco)	10 g/L
bacto trypton (Fa. Difco)	10 g/L
technical casamino acid (Fa. Difco)	10 g/L
$MgSO_4$	0.005 g/L
$FeSO_4$	0.1 g/L
NaCitrat	1.2 g/L
KCl	0.2 g/L
K_2HPO_4	0.4 g/L
L-Cystein	0.6 g/L
Glucose	15 g/L
1,2-$^{13}C_2$-Glucose	2 g/L

[U-$^{13}C_3$]serine Medium T	
brain heart infusion broth (Fa. Difco)	10 g/L
bacto trypton (Fa. Difco)	10 g/L
technical casamino acid (Fa. Difco)	10 g/L
$MgSO_4$	0.05 g/L
$FeSO_4$	0.1 g/L
NaCitrat	1.2 g/L
KCl	0.2 g/L
K_2HPO_4	0.4 g/L
L-Cystein	0.6 g/L
Glucose	15 g/L
U-$^{13}C_3$-serine	0.3 g/L

[U-$^{13}C_3$]glycerol Medium T	
brain heart infusion broth (Fa. Difco)	10 g/L
bacto trypton (Fa. Difco)	10 g/L
technical casamino acid (Fa. Difco)	10 g/L
$MgSO_4$	0.05 g/L
$FeSO_4$	0.1 g/L
NaCitrat	1.2 g/L
KCl	0.2 g/L
K_2HPO_4	0.4 g/L
L-cysteine	0.6 g/L
glucose	15 g/L
U-$^{13}C_3$-glycerol	2.5 g/L

Supplemental Table S27 Retention time and mass fragments of derivatized metabolites used for isotopologue calculations.

Metabolite	Retention time [min]	[M-57]+	[M-85]+
Ala	6.6	m/z 260	
Gly	6.9	m/z 246	
Val	8.4	m/z 288	
Leu	9.0		m/z 274
Ile	9.5		m/z 274
Pro	10.1		m/z 285
Met	13.0	m/z 320	
Ser	13.3	m/z 390	
Thr	13.7	m/z 404	
Phe	14.7	m/z 336	
Asp	15.5	m/z 418	
Glu	17.0	m/z 432	
Lys	18.3	m/z 431	
His	20.2	m/z 440	
Tyr	21.2	m/z 466	

Metabolite	Retention time [min]	[M-57]+	[M-85]+
Lactate	17.6	m/z 261	
Glycolic acid	18.2	m/z 247	
Alanine	19.3	m/z 260	
Glycine	20.2	m/z 246	
Oxalic aicd	20.9	m/z 261	
3-Hydroxy butyric acid	21.4	m/z 275	
Valine	23.6	m/z 288	
Leucine	25.2		m/z 274
Isoleucine	26.3		m/z 274
Succinate	27.3	m/z 289	
Fumarate	28.4	m/z 287	
Glycerol	31.5	m/z 377	
Methionine	34.2	m/z 320	
Serine	35.0	m/z 390	
Threonine	35.9	m/z 404	
Phenyl alanine	37.9	m/z 336	
Malate	38.4	m/z 419	
Aspartate	40.1	m/z 418	
Glutamate	43.5	m/z 432	
Palmitate	43.9	m/z 313	
Oleic acid	48.8	m/z 339	
Stearate	49.5	m/z 341	
Citrate	53.5	m/z 591	

Metabolite	Retention time [min]	[M-15]+	[M-176]+
Gla-1	31.7	m/z 452	
Gla-2	32.8	m/z 452	
Mur-1	35.9		m/z 434
Mur-2	36.4		m/z 434

Metabolite	Retention time [min]	[M-15]+
Glucose in glycogen	8.0	m/z 287

Metabolite	Retention time [min]	[M-15]+
Fructose	33.8	m/z 554
